# CircSEMA4B inhibits the progression of breast cancer by encoding a novel protein SEMA4B-211aa and regulating AKT phosphorylation

**DOI:** 10.1038/s41419-022-05246-1

**Published:** 2022-09-17

**Authors:** Xuehui Wang, Wei Jian, Qifeng Luo, Lin Fang

**Affiliations:** Department of Thyroid and Breast Surgery, Shanghai Tenth People’s Hospital, Tongji University School of Medicine, Shanghai, 200072 China

**Keywords:** Breast cancer, Tumour-suppressor proteins

## Abstract

PI3K/AKT signaling pathway plays an important role in regulating the tumorigenesis, recurrence, and metastasis of breast cancer (BC). In this study, we discovered a circRNA with protein-coding potential, which we named circSEMA4B. CircSEMA4B could encode a novel protein, SEMA4B-211aa. Both circSEMA4B and SEMA4B-211aa were remarkably downregulated in BC tissues and cell lines. Low expression of circSEMA4B was positively associated with TNM stage, tumor size, lymph node metastasis, and distant metastasis of BC patients. The functional investigation showed that circSEMA4B and SEMA4B-211aa could significantly inhibit the proliferation and migration of BC in vivo and in vitro. Of note, SEMA4B-211aa inhibited the generation of PIP3 by binding to p85, thereby inhibiting the phosphorylation of AKT (Thr308). CircSEMA4B inhibited the phosphorylation of AKT (Ser473) through miR-330-3p/PDCD4 axis. Taken together, circSEMA4B is a novel negative regulator of PI3K/AKT signaling pathway, providing novel mechanistic insights into the underlying mechanisms of BC.

## Introduction

Breast cancer (BC) is known as the most common malignancy among women worldwide [1, 2]. In the past few decades, despite leaps of diagnosis and treatment therapies for BC have been achieved, the mortality rates of BC still high due to high incidence of recurrence, distant metastasis, and chemotherapy resistance [3]. Therefore, in-depth investigation focusing on the molecular mechanisms of BC pathogenesis and metastasis is emerging as an urgent issue.

PI3K/AKT signaling pathway plays a central role in BC oncogenic signaling. In up to 70% of human BC, PI3K/AKT signaling pathway was found frequently dysregulated [4]. As one of the most frequently mutated pathways, PI3K/AKT pathway is a critical regulator in multiple cellular processes including cell survival, growth, proliferation, and motility [5], making it an attractive therapeutic target. Therefore, much effort was made to develop targeted therapy for PI3K/AKT signaling pathway in BC [6, 7].

Circular RNAs (circRNAs) is a class of non-coding RNAs (ncRNAs) characterized by a closed-loop structure without 5′ cap or 3′ poly(A) tail, providing them higher ability to resist to RNase and environmental degradation [8–11]. Therefore, circRNAs are more stable in tissues, blood, and exosome compared with conventional linear RNAs [8, 12, 13]. The highly stable nature and remarkable tissue specificity of circRNAs endow them as reliable biomarkers for disease diagnosis and treatment [14, 15]. Previous studies have demonstrated that most circRNAs were originated from exonic and mainly located in the cytoplasm [16]. This type of circRNA typically participated in BC tumorigenesis through acting as miRNA sponges and binding to RNA binding protein (RBP) [17–19]. For example, hsa_circ_0005273 regulates the miR-200a-3p/YAP1 axis and inactivates hippo signaling pathway to promote BC progression [20]. Circ-Foxo3 could bind to both p53 and MDM2, facilitating the addition of ubiquitin to p53 by MDM2 in BC [21]. A small number intron-retaining circRNAs mostly reside in the nuclei and usually function as positive regulators of parent gene transcription [22]. CircRNAs, as a class of ncRNAs, were previously thought to have no ability to encode proteins. However, recent studies revealed that some cytoplasmic circRNAs, which containing open reading frames (ORFs), could encode functional peptides or proteins [23]. Two mechanisms for translation initiation were found to promote 5′-cap-independent translation. The first one is the presence of an Internal Ribosome Entry Site (IRES) [24–26]. Another is the presence of N^6^-methyladenosine (m^6^A) residues [27]. However, up until now, seldom protein-coding circRNA was found involved in BC tumorigenesis [28].

In this study, we found for the first time that circSEMA4B, which was produced by the Semaphorin 4B (SEMA4B) gene, was significantly downregulated in BC tissues and cell lines. Through bioinformatics analysis and mechanism investigations, we verified that a 211-amino acid (aa) protein was encoded by circSEMA4B, which we termed “SEMA4B-211aa”. The functional investigation showed that both circSEMA4B and SEMA4B-211aa exert as tumor suppressors in BC. Of note, circSEMA4B inhibited the activation of PI3K/AKT signaling pathway by encoding the novel protein SEMA4B-211aa and sponging miR-330-3p, indicating it function as a valuable therapeutic target for BC treatment.

## Results

### Characterization of circSEMA4B in BC tissues and cell lines

Increasing evidence has indicated the involvement of circRNAs and PI3K/AKT-related signaling in the process of BC tumorigenesis and progression. In the PathCards (https://pathcards.genecards.org/), 92 associated genes were shown in the PI3K/AKT signaling superpath (Supplementary Table S1). We next analyzed all circRNAs derived from the above genes using circbase (http://circrna.org/) and found 58 circRNAs produced in MCF-7 cells (Supplementary Table S1). According to circRNA Db database (http://reprod.njmu.edu.cn/cgi-bin/circrnadb/circRNADb.php), we next screened out 7 circRNAs with protein-coding capacity. To preliminarily test the differently expressed circRNAs, we examined the expression of candidate circRNAs in randomly selected 15 paired BC tissues and adjacent normal tissues. The results showed that hsa_circ_0000650, we termed circSEMA4B, was significantly lower expressed in 15 BC tissues (Supplementary Fig. S1). Therefore, we chose circSEMA4B for further investigation.

**Fig. 1:**
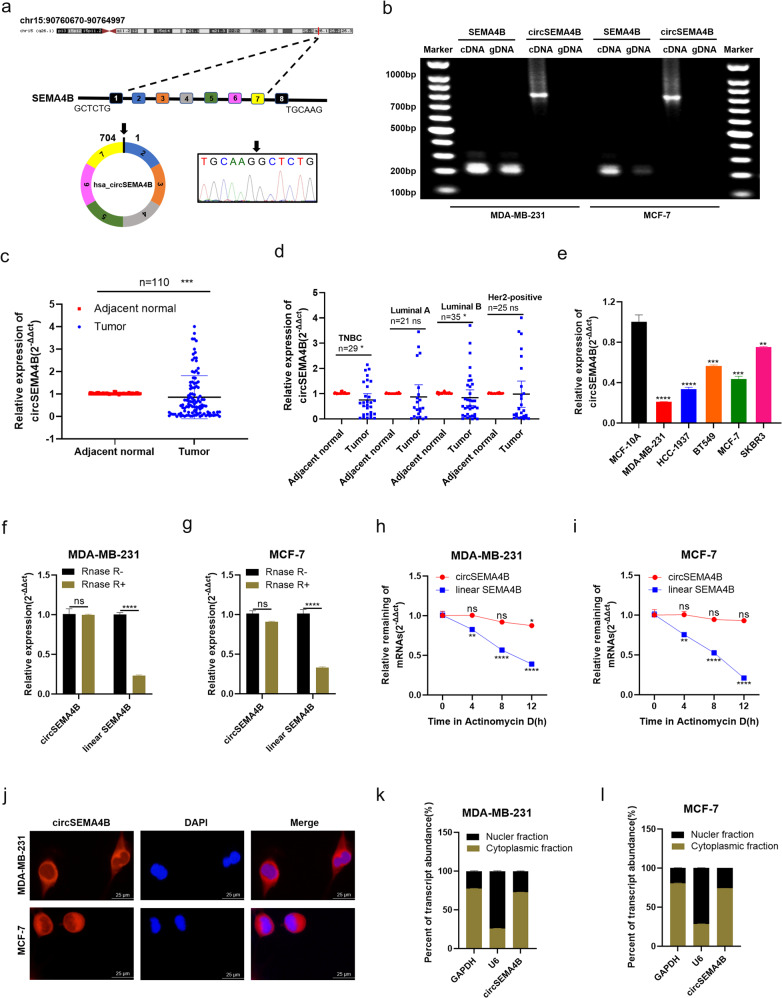
Characterization of circSEMA4B in BC tissues and cell lines. **a** circSEMA4B is formed by circularization of exon 2–7 of the gene SEMA4B, and the splicing junction was verified by Sanger sequencing. **b** Existence of circSEMA4B in BC cells was verified by agarose gel electrophoresis. **c** circSEMA4B had low expression in tumor tissues compared with adjacent normal tissues. **d** Expression of circSEMA4B in TNBC cohort, Her2-positive cohort, luminal-A, and luminal-B cohort, respectively. **e** circSEMA4B had low expression in BC cell lines compared with MCF-10A. **f**, **g** qRT-PCR analysis of hsa_circ_0005273 and linear SEMA4B in BC cells treated with RNase R. **h**, **i** After Actinomycin D treatment, the mRNA stability of circSEMA4B and SEMA4B in BC cells was determined by qRT-PCR. **j** RNA FISH for circSEMA4B and nuclei was stained with DAPI. Red, circSEMA4B; blue, DAPI. **k**, **l** Expression levels of cytoplasmic control transcripts (GAPDH), the nuclear control transcript (U6), and circSEMA4B were determined by qRT-PCR in the cytoplasmic and nuclear fractions of BC cells.

According to the UCSC Genome Browser Home (http://genome.ucsc.edu/), circSEMA4B was generated from exon 2–7 of SEMA4B gene with a full length of 704 bp through a back-splicing way. The junction site of circSEMA4B was confirmed by Sanger sequencing (Fig. 1a). The specificity of circSEMA4B PCR products from BC cell lines were verified by agarose gel electrophoresis. SEMA4B could be amplified by primers in both cDNA and gDNA, but circSEMA4B could only be amplified by primers in cDNA but not gDNA (Fig. 1b). In order to evaluate the biological importance of circSEMA4B in BC, we examined circSEMA4B expression in 110 BC tissues and adjacent normal tissues and found that circSEMA4B has significantly low expression levels in BC tissues (Fig. 1c). As BC is well documented for the clear classification of 4 molecular subtypes, including TNBC, Her2-Positive, luminal-A, and luminal-B. We further explore whether CircSEMA4B is indiscriminately deregulated in all subtypes of breast cancer. We found that circSEMA4B was significantly high expressed in TNBC cohort and luminal-B cohort (Fig. 1d). Moreover, circSEMA4B expression was decreased in BC cell lines compared to the normal breast epithelial cell line MCF-10A, especially in TNBC cell line MDA-MB-231 and luminal cell line MCF-7 (Fig. 1e). Therefore, we chose to explore the function and mechanism of circSEMA4B in MDA-MB-231 and MCF-7 cell lines. To further identify the potential role of circSEMA4B as biomarker in BC, relationship between circSEMA4B expression and several clinical pathological variables in 110 BC patients was analyzed. As shown in Table 1, low expression of circSEMA4B was positively associated with TNM stage, tumor size, lymph node metastasis, recurrence, and metastasis, but had no correlation with age. Next, we explored the characteristics and cellular localization of circSEMA4B in BC cells. Treatment of RNase R did not alter the circSEMA4B level, while SEMA4B mRNA level reduced significantly (Fig. 1f, g). Actinomycin D assay was also conducted and showed that circSEMA4B rather than SEMA4B could resist to Actinomycin D, indicating circSEMA4B was more stable and had a longer half-life time compared with the linear form of SEMA4B mRNA (Fig. 1h, i). To determine circSEMA4B localization, we performed FISH assay in MDA-MB-231 and MCF-7 cell lines, showing circSEMA4B mainly displayed a cytoplasmic localization (Fig. 1j). Consistently, through subsequent qRT-PCR analysis of cell fractions, circSEMA4B predominantly localized in the cytoplasm rather than nuclear (Fig. 1k, l).

**Table 1:** The relationship between the expression of circSEMA4B and various clinicopathological variables.

Patients characteristics	Total	circSEMA4B expression		
		High (*N* = 31)	Low (*N* = 79)	*P* value*
Age				0.1242
<60	31	12	19	
≥60	79	19	60	
TNM stage				0.0002***
I and II	74	29	45	
III and IV	36	2	34	
Tumor size (cm)				0.0001****
≤2	30	21	9	
>2	80	10	70	
Lymph node metastasis				0.0001****
Negative	59	27	32	
Positive	51	4	47	
Recurrence and metastasis				0.0321*
No	99	31	68	
Yes	11	0	11	

*P < 0.05, ***P < 0.001, ****P < 0.0001.

### CircSEMA4B suppresses the proliferation, migration, and invasion abilities of BC cells

**Fig. 2:**
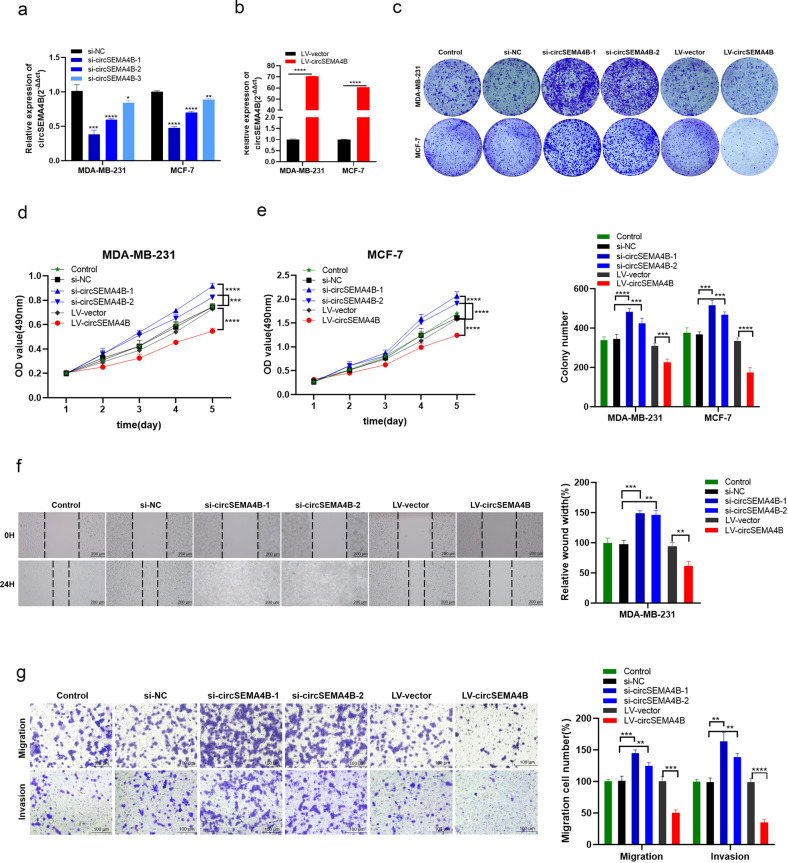
CircSEMA4B suppresses the proliferation, migration, and invasion abilities of BC cells. **a** Expression of circSEMA4B was confirmed by qRT-PCR in BC cells transfected with si-NC or si-circSEMA4B. **b** Expression of circSEMA4B was confirmed by qRT-PCR in BC cells transfected with LV-vector or LV-circSEMA4B. **c** Effect of circSEMA4B on proliferation in BC cell lines by colony formation assay. **d**, **e** Effect of circSEMA4B on proliferation in BC cell lines by MTT assay. **f** Wound healing assays were performed in MDA-MB-231 treated with si-circSEMA4B or LV-circSEMA4B. **g** Cell migration assays and invasion assays were performed in MDA-MB-231 treated with si-circSEMA4B or LV-circSEMA4B using transwell chambers.

To investigate the potential role of circSEMA4B in BC cells, we designed 3 specific siRNA targeting the back-splice junction sequence of circSEMA4B (si-circSEMA4B) to inhibit its expression and si-NC as a control, and the silencing efficacy was shown in Fig. 2a. Meanwhile, we stably overexpressed circSEMA4B in BC cells using specific circSEMA4B lentiviral plasmid (LV-circSEMA4B) and LV-vector as a control (Fig. 2b). Results from MTT assay and colony formation assay demonstrated that circSEMA4B remarkably suppressed BC cells proliferation (Fig. 2c–e). Furthermore, depletion of circSEMA4B promoted BC cells migration compared to the controls while circSEMA4B overexpression significantly led to decreased cell migration in MDA-MB-231 cells (Fig. 2f, g). In addition, the invasion ability of MDA-MB-231 cells also increased after suppressing circSEMA4B expression (Fig. 2g). All results above demonstrated that circSEMA4B act as a tumor suppressor in BC cells.

### CircSEMA4B encodes a novel protein, SEMA4B-211aa

**Fig. 3:**
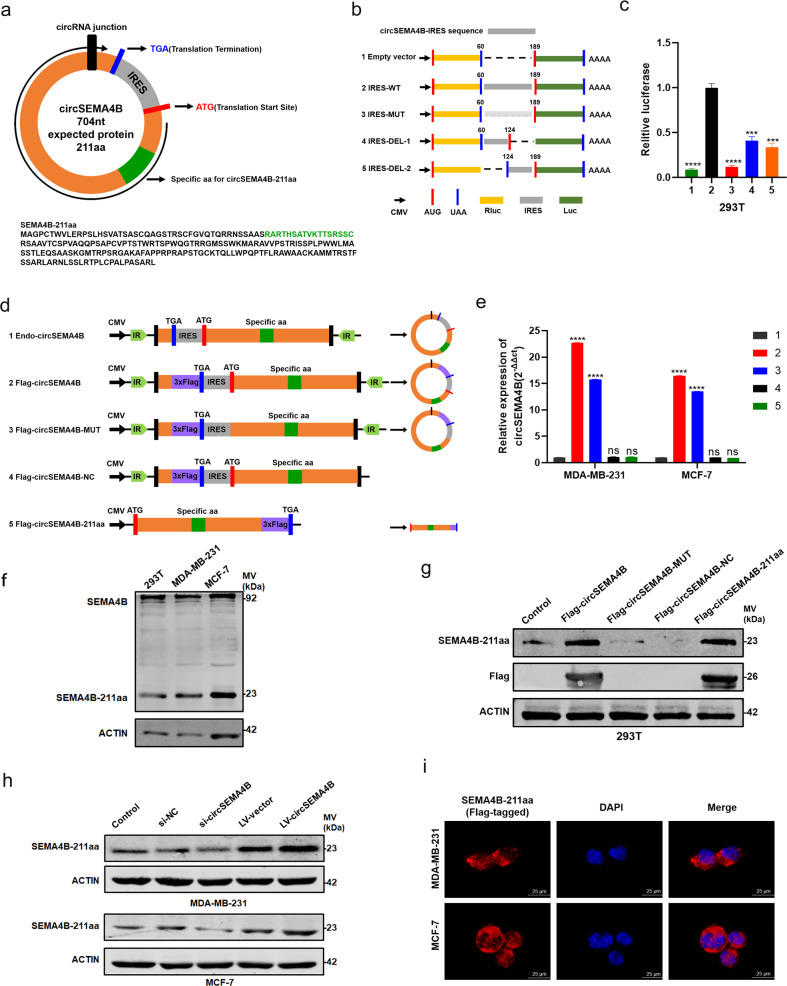
CircSEMA4B encodes a novel protein, SEMA4B-211aa. **a** Upper panel, the putative ORF in circSEMA4B. Lower panel, the sequences of the putative ORF in circSEMA4B. **b** IRES sequences in circSEMA4B or its different truncations were cloned between Rluc and Luc reporter genes with independent start and stop codons. **c** The relative luciferase activity in the above vectors was tested. **d** Vectors set for detecting circSEMA4B encoded protein. **e** Expression of circSEMA4B was detected in BC cells transfected with Flag-circSEMA4B, Flag-circSEMA4B-MUT, Flag-circSEMA4B-NC, and Flag-circSEMA4B-211aa. **f** Anti-SEMA4B-211aa were used to detect SEMA4B-211aa and SEMA4B protein. **g** Anti-FLAG and anti-SEMA4B-211aa were used to detect SEMA4B-211aa in 293T cells transfected with Flag-circSEMA4B, Flag-circSEMA4B-MUT, Flag-circSEMA4B-NC, and Flag-circSEMA4B-211aa. **h** Expression of circSEMA4B-211aa in BC cells transfected with si-circSEMA4B or LV-circSEMA4B. **i** Flag-tagged SEMA4B-211aa was transfected into BC cells. IF staining using anti-Flag was performed to show the SEMA4B-211aa cellular localization. Red, SEMA4B-211aa; blue, DAPI.

Recent years, emerging evidence has indicated that some circRNAs have the protein-coding capacity. As annotated in circRNA Db database (http://reprod.njmu.edu.cn/cgi-bin/circrnadb/circRNADb.php), circSEMA4B had putative IRES sequence and a 636-nt ORF, indicating it might encode a 211aa novel protein, which was termed “SEMA4B-211aa” in this study (Fig. 3a). The expected size of SEMA4B-211aa was about 23 kDa. We first tested the activity of the predicted IRES in circSEMA4B by dual-luciferase assay. IRES sequences in circSEMA4B or its different truncations were cloned on pcDNA 3.1(+) vector between Rluc and Luc reporter genes with independent start codon AUG and stop codon UAA (Fig. 3b). The luciferase assay showed that the full-length IRES induced the highest Luc/Rluc activity compared with the truncated IRES and the mutated IRES generated by restriction cloning. In contrast, empty vectors could not induce Luc activation, indicating that this IRES could induce 5′-cap-independent translation (Fig. 3c). Next, we established a set of vectors to confirm that circSEMA4B is translatable in human cells. As shown in Fig. 3d, the junction of endogenous circSEMA4B is inside the ORF (endo-circSEMA4B). We added the 3xFLAG sequence before the stop codon of the ORF (Flag-circSEMA4B). Downstream flanking sequences were deleted (Flag-circSEMA4B-NC) for a negative control, and the linearized ORF added FLAG-tag was cloned in a linear vector (Flag-circSEMA4B-211aa) for a positive control. To exclude the possibility that the biological functions were induced by the encoded protein instead of circSEMA4B, we constructed circSEMA4B with stop codon ATG deletion plasmid, which could form circSEMA4B RNA structure but could not be translated (Flag-circSEMA4B-MUT). As expected, through transfecting above plasmids into MDA-MB-231 and MCF-7 cells, the mRNA level of circSEMA4B was successfully overexpressed in both Flag-circSEMA4B and Flag-circSEMA4B-MUT group rather than Flag-circSEMA4B-211aa and Flag-circSEMA4B-NC group (Fig. 3e). To prove the presence of the translated endogenous protein SEMA4B-211aa, we constructed an antibody specifically targeting the putative circSEMA4B translated protein, anti-SEMA4B-211aa. Through analyzing the specificity, hydrophilicity, and immunogenicity of the predicted translated peptide, the RARTHSATVKTTSRSSC segment was chosen as the antigenic region for the preparation of specific antibodies (antigen design shown in Fig. 3a, lower panel). The concentration and titer of the specific antibody have been verified by Dot Immunobinding Assay provided by Abclonal (Supplementary Fig. S2a). SEMA4B-211aa expression was identified in non-transfected 293T, MDA-MB-231, and MCF-7 cell lines by western blotting at the predicted molecular weight. Also, anti-SEMA4B-211aa could recognize SEMA4B protein at 92 kDa since SEMA4B-211aa and SEMA4B protein shared the same specific segment (Fig. 3f). Then we transfected plasmids shown in Fig. 3d into 293T cells and detected their potential translated products. The FLAG-tag antibody only detected an ~26 kDa protein in Flag-circSEMA4B and Flag-circSEMA4B-211aa transfected cells, indicating that the Flag-circSEMA4B is translated. Anti-SEMA4B-211aa could detect endogenous SEMA4B-211aa in both transfected and non-transfected cells, while the Flag-circSEMA4B and Flag-circSEMA4B-211aa transfected cells showed obvious overexpression of SEMA4B-211aa (Fig. 3g). Furthermore, The SEMA4B-211aa protein level was positively regulated by circSEMA4B in BC cells (Fig. 3h). As SEMA4B-211aa is formed by the “spanning junction ORF,” the identified distinctive amino acids in this region further suggested that this novel protein was encoded by circSEMA4B rather than SEMA4B. Given that the presence of m^6^A residues also has the ability to initiate circRNAs translation [27]. Moreover, m^6^A modification may enhance the translational efficacies in some specific circRNAs [29, 30]. Therefore, we further explore whether m^6^A modification affect the generation of SEMA4B-211aa. According to the analysis in SRAMP, two “very high confidence” m^6^A modification sites were predicted in circSEMA4B sequence (Supplementary Fig. S2b–d). Then methylated RNA immunoprecipitation (MeRIP) followed by qRT-PCR were performed to detect the m^6^A modification of circSEMA4B. However, circSEMA4B pulled down in anti-m^6^A group were not higher than in anti-IgG group, indicating that circSEMA4B translation was not influenced by m^6^A modification (Supplementary Fig. S2e). To investigate the molecular mechanism that SEMA4B-211aa involved in, we examined the localization of SEMA4B-211aa by IF. As shown in Fig. 3i, exogenously transfected flag-tagged SEMA4B-211aa mainly localized in cytoplasm of BC cells.

### SEMA4B-211aa acts as a tumor suppressor in BC cells

**Fig. 4:**
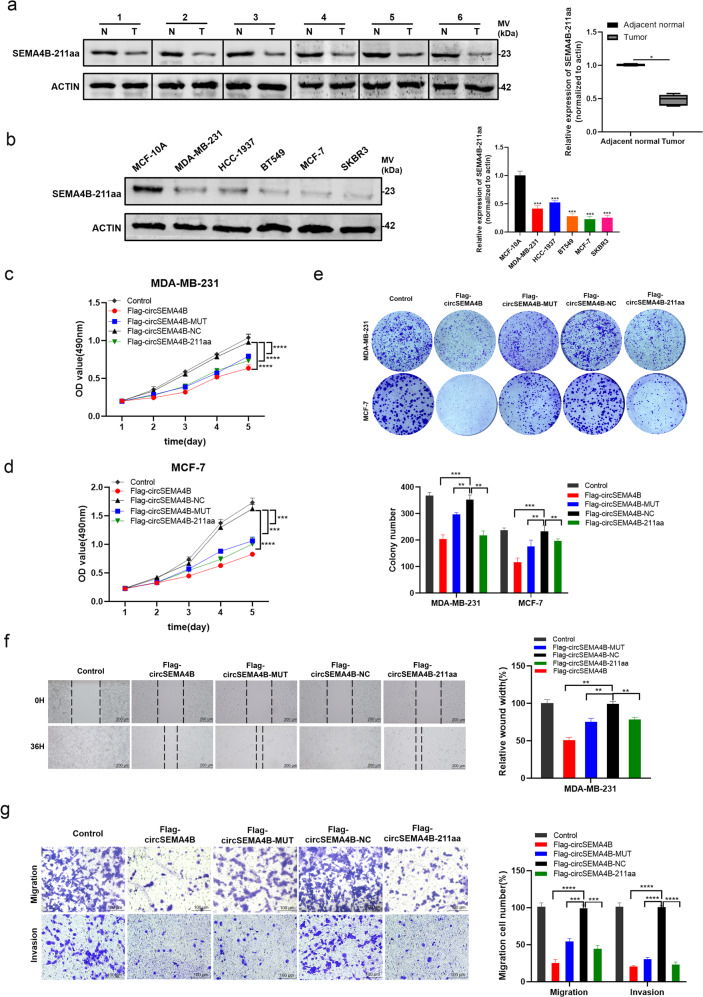
SEMA4B-211aa acts as a tumor suppressor in BC cells. **a** SEMA4B-211aa had low expression in BC tissues compared with adjacent normal tissues. **b** SEMA4B-211aa had low expression in BC cell lines compared with MCF-10A. **c**, **d** Effect of SEMA4B-211aa on proliferation in BC cell lines by MTT assay. **e** Effect of SEMA4B-211aa on proliferation in BC cell lines by colony formation assay. **f** Effect of SEMA4B-211aa on migration in MDA-MB-231 by wound healing assays. **g** Effect of SEMA4B-211aa on migration and invasion in MDA-MB-231 by transwell assays.

We examined SEMA4B-211aa expression in BC, and found that SEMA4B-211aa was lowly expressed in BC tissues and cell lines, indicating it a tumor suppressor in BC (Fig. 4a, b). As circSEMA4B could encode SEMA4B-211aa, it is necessary to further study whether circSEMA4B itself or the encoded protein SEMA4B-211aa exert the tumor suppressor role in BC. Therefore, we transfected Flag-circSEMA4B, Flag-circSEMA4B-MUT, Flag-circSEMA4B-NC, and Flag-circSEMA4B-211aa plasmids into BC cells to explore the biological functions of SEMA4B-211aa and circSEMA4B itself. Similar with the previous results (Fig. 2c–g), overexpression of circSEMA4B with flag labeling act as a tumor suppressor in BC cells. Moreover, the linear SEMA4B-211aa, encoded by circSEMA4B, could also significantly inhibit BC cells proliferation by MTT assay and colony formation assay (Fig. 4c–e), as well as migration and invasion by wound healing assay and tanswell assay (Fig. 4f, g). It is a remarkable fact that overexpression of mutant circSEMA4B with flag labeling, which was unable to translate SEMA4B-211aa, could also decrease the proliferation, migration, and invasion ability of BC cells (Fig. 4c–g). The above results showed that both circSEMA4B itself and SEMA4B-211aa exert the tumor suppressor roles in BC, indicating that circSEMA4B could inhibit the progression of BC through at least two ways.

### SEMA4B-211aa interacts with p85 and regulates PI3K activity

**Fig. 5:**
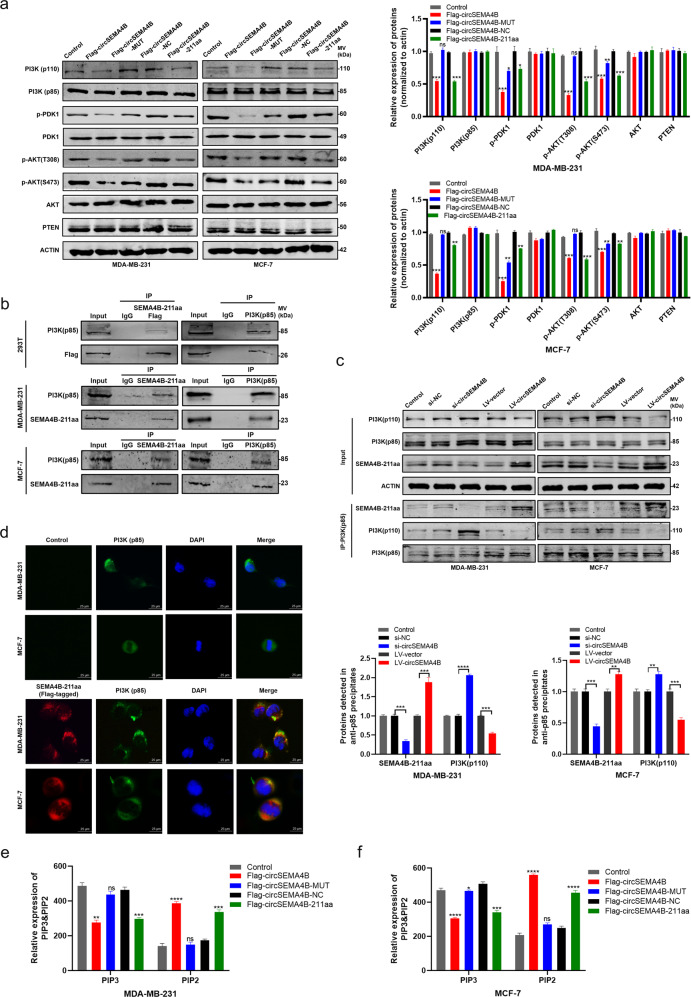
SEMA4B-211aa interacts with p85 and regulates PI3K activity. **a** Expression of key proteins in PI3K pathway were detected in BC cells transfected with Flag-circSEMA4B, Flag-circSEMA4B-MUT, Flag-circSEMA4B-NC, and Flag-circSEMA4B-211aa. **b** Mutual interaction of p85 and SEMA4B-211aa were determined by Co-IP. **c** p85/p110 complex was influenced by SEMA4B-211aa by Co-IP. **d** Colocalization of p85 and SEMA4B-211aa in BC cells. Red, SEMA4B-211aa; green, p85; blue, DAPI. **e**, **f** PI3K activity was determined by ELISA according to PIP2/PIP3 expression in BC cells transfected with Flag-circSEMA4B, Flag-circSEMA4B-MUT, Flag-circSEMA4B-NC, and Flag-circSEMA4B-211aa.

As we have demonstrated that circSEMA4B was produced from PI3K/AKT signaling-associated genes, we tried to explore the role of SEMA4B-211aa in regulating PI3K/AKT pathway. The key protein levels of PI3K/AKT pathway were examined by Western blot. As shown in Fig. 5a, SEMA4B-211aa did not affect the protein level of total PDK1 and AKT. Instead, SEMA4B-211aa significantly inhibited the phosphorylation of PDK1 and AKT-Thr308, while the protein level of PTEN was not affected after both SEMA4B-211aa and circSEMA4B overexpression, indicating that SEMA4B-211aa could attenuate PDK1 and AKT phosphorylation independently from PTEN. More interestingly, though the regulatory subunit p85 was not affected by both circSEMA4B and SEMA4B-211aa, the catalytic subunits p110 decreased induced by SEMA4B-211aa rather than circSEMA4B. In addition, we investigated whether circSEMA4B affect the expression of SEMA4B. The results showed that there was no significant change in mRNA and protein level of SEMA4B after knockdown and overexpression of circSEMA4B, indicating circSEMA4B exert its roles independent of its parent gene (Supplementary Fig. S3a, b). Taking all results above, we inferred that SEMA4B-211aa exerted its effects upstream of p110. Then, we examined whether SEMA4B-211aa reduced the expression of p110 at the transcriptional level (Supplementary Fig. S3c). Unlike the protein levels, the mRNA levels of p110 were not affected by SEMA4B-211aa, showing that the reduction of p110 protein occurred at the post-transcriptional stage. To explore the SEMA4B-211aa-interacted candidates, silver staining assay was performed, and specific bands were enriched at 70–100 kD in both 293T (Supplementary Fig. S3d). Given that regulatory subunit p85 was a upstream signal to stabilize catalytic subunits p110 [31], we inferred that p85 was a potential binding protein to SEMA4B-211aa. In 293T cells, Flag-tagged SEMA4B-211aa had mutual interaction with p85 confirmed by Co-IP (Fig. 5b). Consistently, we further verified the interaction between SEMA4B-211aa and p85 in MDA-MB-231 and MCF-7 cells under physiological condition by specific antibody against SEMA4B-211aa, indicating the interaction between SEMA4B-211aa and p85 in vivo. Given that p85 could exist as a monomer or in the heterodimeric complex with p110, so we further examined whether p110 is present in the complex of SEMA4B-211aa and p85. However, p110 could not be detected in the anti-SEMA4B-211aa precipitates (Supplementary Fig. S3e), indicating that it is free p85, rather that the p85/p110 complex interacts with SEMA4B-211aa. Furthermore, we further found that the amount of SEMA4B-211aa could influence p85/p110 complex. SEMA4B-211aa was downregulated or upregulated in BC cells, followed by immunoprecipitation of SEMA4B-211aa, p85, or p110. Less p110 was detected in anti-p85 precipitates in SEMA4B-211aa overexpression BC cells, while more p110 was detected in SEMA4B-211aa knocked down BC cells (Fig. 5c). In addition, the purified GST-tagged SEMA4B-211aa could not pull down His-tagged PI3K(p85) in vitro binding assay, further indicating that SEMA4B-211aa interacted with PI3K(p85) indirectly (Fig. S3f). Moreover, IF staining in MDA-MB-231 and MCF-7 cells transfected with Flag-tagged SEMA4B-211aa also supported the colocalization of SEMA4B with p85 (Fig. 5d). Furthermore, the PI3K activity was determined by ELISA according to conversion of PIP2 lipid to PIP3 lipid, and the results showed that SEMA4B-211aa significantly inhibited PIP3 generation (Fig. 5e, f). Taking all results above, SEMA4B-211aa may compete with p110 to bind to p85, thereby decreasing the stability of p110 and suppressing the generation of second messenger PIP3.

### CircSEMA4B regulates PDCD4 and PI3K/AKT pathway through acting as a sponge for miR-330-3p

**Fig. 6:**
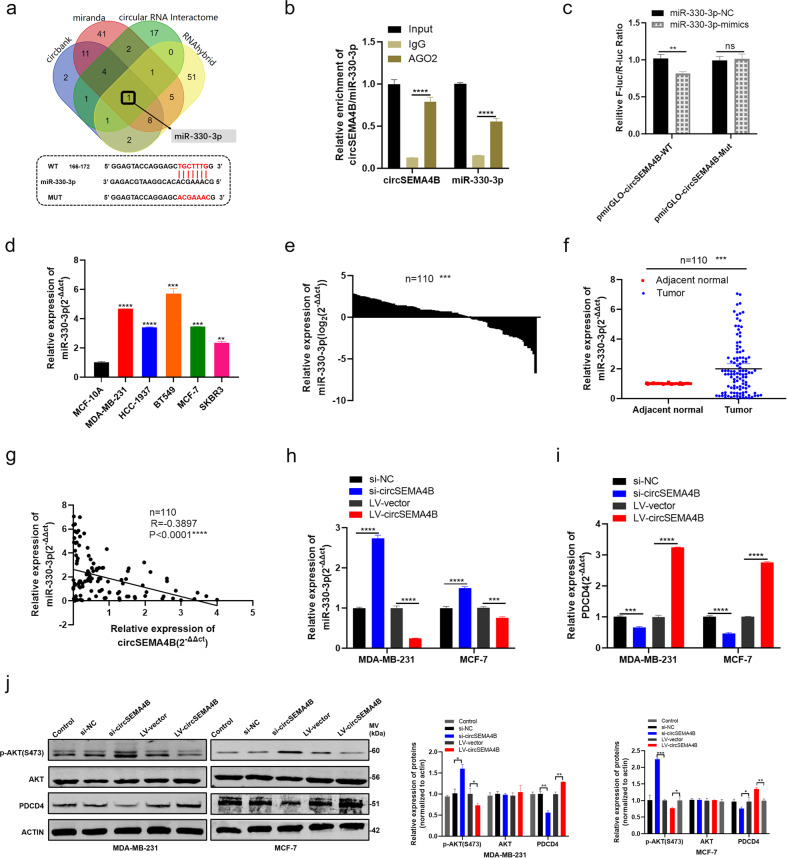
CircSEMA4B serves as a sponge for miR-330-3p. **a** Upper panel, Venn diagram showing the potential target miRNAs of circSEMA4B. Lower panel, Putative complementary sites within miR-330-3p and circSEMA4B predicted by bioinformatics analysis. **b** RIP experiments were performed in HEK293T cells, and the co-precipitated RNA was subjected to qRT-PCR for miR-330-3p and circSEMA4B. **c** Dual-luciferase reporter assays demonstrated that miR-330-3p is a direct target of circSEMA4B. **d** miR-330-3p had high expression in BC cell lines compared with MCF-10A. **e**, **f** miR-330-3p had high expression in BC tissues compared with adjacent normal tissues. **g** Correlations between the expression of circSEMA4B and miR-330-3p were found with Pearson’s correlation analysis in BC tissue samples (*n* = 110). **h** Expression of miR-330-3p in BC cells transfected with si-circSEMA4B or LV-circSEMA4B. **i** Expression of PDCD4 in BC cells transfected with si-circSEMA4B or LV-circSEMA4B. **j** The protein levels of PDCD4 and AKT/p-AKT(S473) in BC cells transfected with si-circSEMA4B or LV-circSEMA4B.

As we have demonstrated that both circSEMA4B and SEMA4B-211aa could significantly inhibit the proliferation and migration of BC cells, we further explore the underlying mechanisms of circSEMA4B. According to the previous studies, circRNAs mainly located in the cytoplasm have been reported serve as miRNA sponge in an AGO2 manner [32], we speculated that circSEMA4B might inhibit BC cells tumorigenesis via sponging some specific miRNAs. Then, Starbase (http://starbase.sysu.edu.cn/index.php), CircInteractome (https://circinteractome.nia.nih.gov/), miranda, and RNAhybird were employed to find the potential miRNAs, and Venn diagram was used for further analysis. As shown in Fig. 6a, there was a complementary base sequence between miR-330-3p and circSEMA4B. To validate whether circSEMA4B could directly target miR-330-3p, we first performed RIP assay and found that both circSEMA4B and miR-330-3p pulled down in anti-AGO2 group were higher than in anti-IgG group, indicating that circSEMA4B have the possibility to sponge miR-330-3p (Fig. 6b). Then, luciferase reporters was conducted and results showed that miR-330-3p could significantly inhibited the luciferase reporter activity compared to the control group (Fig. 6c). We detected miR-330-3p in BC tissues and cells lines by qRT-PCR, and found that miR-330-3p was enriched in BC (Fig. 6d–f). More importantly, miR-330-3p expression was negatively correlated with circSEMA4B in 110 paired BC tissues (Fig. 6g). In our previous studies, miR-330-3p has been found act as an oncogenic role in BC and miR-330-3p upregulation indicated poor prognosis in patients with BC [33, 34]. In addition, miR-330-3p could inhibit PDCD4 through directly interacting with the 3′ UTR of PDCD4 mRNA [35, 36]. Base on the previous studies related to miR-330-3p, we speculated that circSEMA4B could positively regulate PDCD4 via miR-330-3p. The qRT-PCR results in BC cells showed that miR-330-3p expression increased and PDCD4 expression decreased after circSEMA4B was downregulated, and the expression of miR-330-3p and PDCD4 showed opposite changes after circSEMA4B was upregulated (Fig. 6h, i). Since PDCD4 has been reported act as a suppressor on PI3K/AKT signaling pathway and inhibited the phosphorylation of AKT-Ser308 [37, 38], we further examined whether circSEMA4B could affect PI3K/AKT signaling pathway in BC. As expected, after circSEMA4B was downregulated, the protein level of PDCD4 decreased and the protein levels of p-AKT(S473) increased, indicating the activation of PI3K/AKT signaling pathway (Fig. 6j). We further examined whether SEMA4B-211aa influenced the expression of PDCD4, and verified that it was circSEMA4B, rather than SEMA4B-211aa, that could regulate the protein level of PDCD4 (Supplementary Fig. S3g). In addition, depletion of PDCD4 did not affect the expression of SEMA4B-211aa (Supplementary Fig. S3h).

**Fig. 7:**
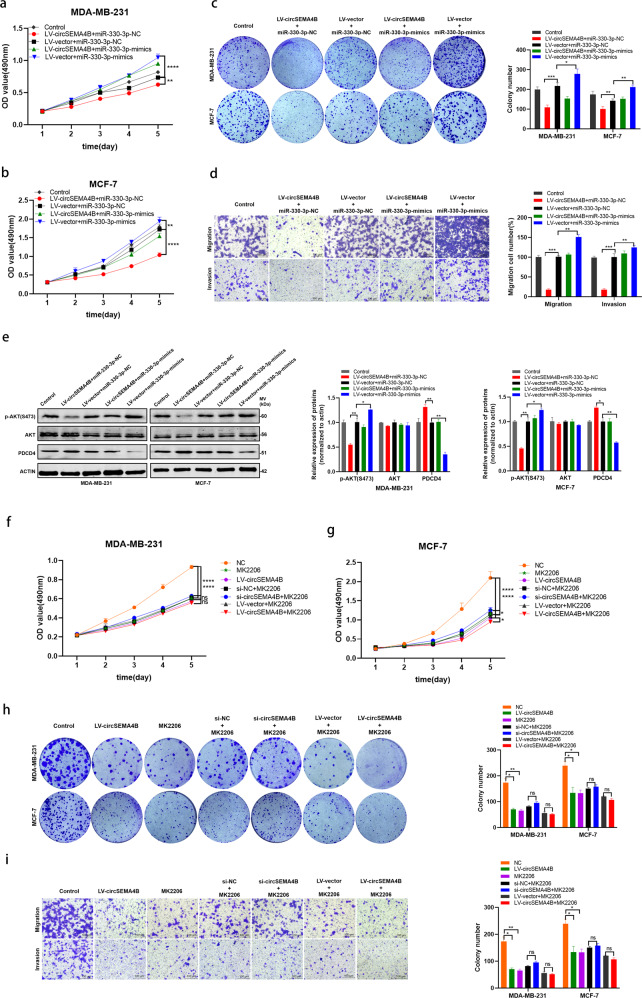
CircSEMA4B regulates PDCD4 and PI3K/AKT pathway through acting as a sponge for miR-330-3p. **a**, **b** miR-330-3p-mimics rescued the suppressive effects of LV-circSEMA4B in BC cells by MTT assay. **c** miR-330-3p-mimics rescued the suppressive effects of LV-circSEMA4B in BC cells by colony formation assay. **d** miR-330-3p-mimics rescued the suppressive effects of LV-circSEMA4B in BC cells by transwell assay. **e** Western blot showed that miR-330-3p-mimics can partly rescue the high expression of PDCD4 and low expression of p-AKT(S473) caused by LV-circSEMA4B in BC cells. **f**, **g** Effect of circSEMA4B on proliferation in BC cell lines treated with MK2206 by MTT assay. **h** Effect of circSEMA4B on proliferation in BC cell lines treated with MK2206 by colony formation assay. **i** Effect of circSEMA4B on migration and invasion in MDA-MB-231 cell line treated with MK2206 using transwell chambers.

To validate circSEMA4B affected BC cells proliferation, migration, invasion and maintained the PDCD4 expression via acting as a sponge for miR-330-3p, we performed rescue assays in MDA-MB-231 and MCF-7 cells. We co-transfected LV-circSEMA4B and miR-330-3p-mimics in BC cells, and found that LV-circSEMA4B induced BC cells proliferation and migration reduction were partly inverted by miR-330-3p-mimics according to MTT assay, colony formation assay and transwell assay (Fig. 7a–d). Furthermore, at the protein level, miR-330-3p mimics partially rescued the upregulation of PDCD4 and the downregulation of p-AKT(S473) induced by LV-circSEMA4B (Fig. 7e).

Taken together, all results above indicated that circSEMA4B inhibits the oncogenic PI3K/Akt pathway via two different ways: encoding a novel protein through IRES-dependent translation and sponging miR-330-3p to promote the expression of its target gene PDCD4. To further identify that circSEMA4B functions as a tumor suppressor mainly through PI3K/Akt pathway, we exploit whether Akt inhibitor MK2206 recapitulates the effect of circSEMA4B overexpression in repressing the malignant phenotypes of BC cells. As expected, the inhibitory effect of circSEMA4B on proliferation (Fig. 7f, g), migration, and invasion (Fig. 7h, i) was significantly weakened in BC cells treated with MK2206, indicating that circSEMA4B specifically targets PI3K/AKT pathway.

### CircSEMA4B suppresses BC tumorigenicity in vivo

**Fig. 8:**
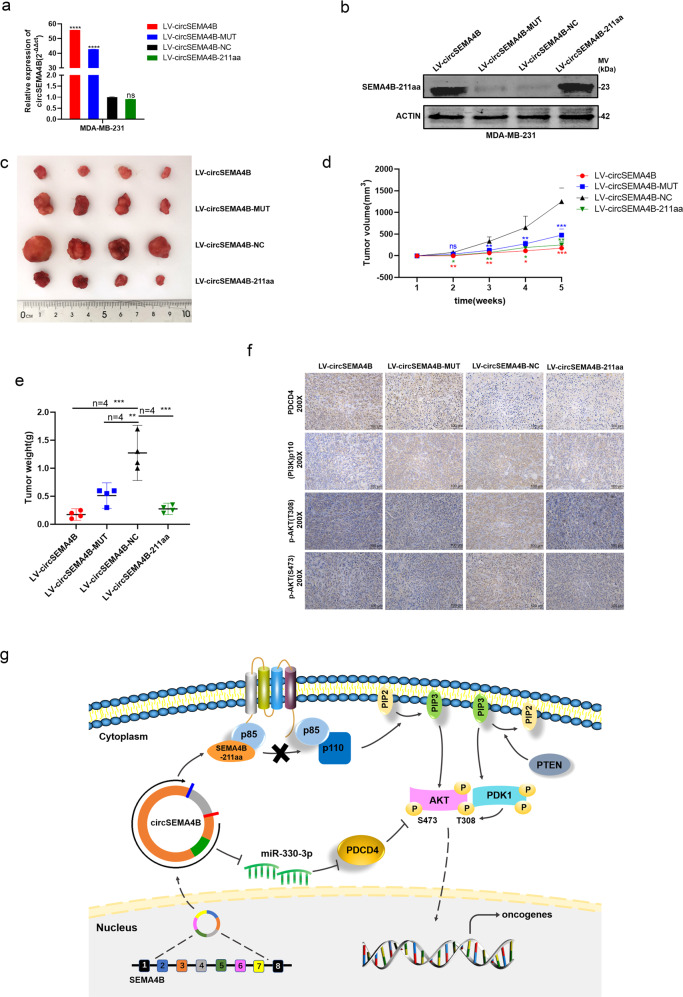
CircSEMA4B suppresses BC tumorigenicity in vivo. **a** The transfection efficiency in MDA-MB-231 cells were examined by qRT-PCR. **b** The transfection efficiency in MDA-MB-231 cells were examined by western blot. **c** Representative images of xenograft tumors in nude mice. **d** The growth curves of xenografts. **e** Average tumor weight of nude mice. **f** Immunohistochemistry (IHC) staining of PDCD4, p110 and p-AKT (T308/S373) in xenografts. **g** The mechanism diagram was generated to illustrate the mechanism of circSEMA4B and SEMA4B-211aa in BC.

To further explore the biological role of circSEMA4B in growth of BC cells in vivo, we generated stable transfection of above-mentioned four vectors in Fig. 3d, which were termed “LV-circSEMA4B, LV-circSEMA4B-MUT, LV-circSEMA4B-NC, and LV-circSEMA4B-211aa” in this study. The transfection efficiency in MDA-MB-231 cells were examined by qRT-PCR and western blot (Fig. 8a, b). Consistent with results in vitro, tumors generated from cells transfected with LV-circSEMA4B, LV-circSEMA4B-MUT, and LV-circSEMA4B-211aa had smaller size (Fig. 8c, d) and lower weight (Fig. 8e) compared to the LV-circSEMA4B-NC group, indicating that overexpression of circSEMA4B and SEMA4B-211aa dramatically attenuated tumor growth. Thereafter, we detected the expression levels of PDCD4, p110, and p-AKT (T308/S373) in xenograft tumors by IHC (Fig. 8f). It is circSEMA4B rather than SEMA4B-211aa overexpression positively correlated with the expression levels of PDCD4. In addition, SEMA4B-211aa overexpression reduced the expression levels of p110. Moreover, both circSEMA4B and SEMA4B-211aa overexpression significantly decreased the expression levels of p-AKT. Together, our study showed that circSEMA4B is a critical negative regulator of PI3K/AKT signaling in BC tumorigenesis through at least two ways: encoding a novel protein SEMA4B-211aa and regulating miR-330-3p/PDCD4 axis (Fig. 8g).

## Discussion

In the past decades, PI3K/AKT signaling pathway has been identified involved in BC tumorigenesis, recurrence, and metastasis [39]. According to their homology and substrate specificity, PI3Ks were grouped into three classes [40], among them class IA PI3K is the most common member implicated in cancer, which were heterodimeric enzymes composed of regulatory subunit P85 and catalytic subunit P110 [41]. Upon receptor tyrosine kinase activation, the p85/p110 complex is recruited to phosphotyrosine residues in the activated receptor via the p85 SH2 domains. This alleviates the inhibitory contacts with the p110 catalytic subunit, while also bringing p110 into close proximity with its lipid substrates. The active PI3K then converts phosphatidylinositol-4,5-bisphosphate (PIP2) to a major second messenger phosphatidylinositol-3,4,5-trisphosphate (PIP3) and triggers a downstream signaling cascade, including the activation of AKT and PDK1 [40, 42]. Previous studies have proved that the phosphorylation of AKT represents the activation of PI3K signaling pathway. The full activation of Akt is a multistep process, but the final step is to phosphorylate Akt on two sites, Thr308 and Ser473, which are necessary and sufficient for the full activation of Akt [43, 44].

In the present study, we identified circSEMA4B was remarkably downregulated in BC tissues and cell lines. Of note, abnormal low expression of circSEMA4B was positively related to the clinical pathological variables in 110 BC patients, indicating it is a potential biomarker. Driven by an active IRES, circSEMA4B encoded a novel protein SEMA4B-211aa. In the previous studies associated with protein-coding circRNAs, researchers showed that it was the encoded proteins, rather than circRNAs regulate the biology activities in cancers [45, 46]. However, the functional investigation showed that both circSEMA4B and SEMA4B-211aa exert as tumor suppressors in BC progression in vivo and in vitro. Therefore, we tried to investigate the molecular mechanisms of circSEMA4B in BC pathogenesis.

Through Co-IP assay, we demonstrated that SEMA4B-211aa inhibit the formation of p85/p110 complexes by forming a complex with p85, leading to a decrease in p110 proteins, presumably due to the well-known instability of p110 monomers. This decrease in p110 proteins results in attenuation of PI3K signaling. Given that p85/p110 complexes is irreversible [47, 48], and results of co-IP indicated that it is P85 rather than P110 could interact with SEMA4B-211aa, indicating that SEMA4B-211aa could indirectly bind to free p85 but not p110 or p85/p110 complex. The decrease of p85/p110 complex inhibit the generation of PIP3, thereby inhibiting the phosphorylation of AKT (Thr308). Further studies will be necessary to identify other potential components in the SEMA4B-211aa -P85 complex and elucidate the full complexity of the role of the complex. On the other hand, circSEMA4B was proved upregulate PDCD4 via acting as miR-330-3p sponge. PDCD4 has been confirmed as a tumor suppressor gene involved in multiple cancer-related process. The inactivation of tumor suppressor PDCD4 was a key rate-limiting step in either the initial or progression stage of BC [49, 50]. Previous studies have showed that PDCD4 could inhibit the phosphorylation of AKT (Ser473) [38, 51]. Therefore, we further demonstrated that circSEMA4B inhibit the phosphorylation of AKT (Ser473) through miR-330-3p/PDCD4 axis. Taken together, circSEMA4B inhibited the phosphorylation of AKT and activation of PI3K/AKT signaling pathway by at least two ways: encoding the novel protein SEMA4B-211aa and sponging miR-330-3p. As a highly potent and selective allosteric Akt inhibitor, MK2206 could suppress BC tumorigenesis through the suppression of phospho-Akt expression and its downstream molecules [52, 53]. We identify that tumor suppressive role of circSEMA4B was significantly weakened in BC cells treated with Akt inhibitor MK2206, further showing that Akt activation is predominantly involved in the tumor suppressive role of circSEMA4B in BC.

In summary, circSEMA4B was a newly identified protein-coding circRNA. Both circSEMA4B and the novel encoded protein SEMA4B-211aa were expressed at low levels in BC, and exerted as tumor suppressors in vivo and in vitro. SEMA4B-211aa inhibited the generation of PIP3 by competing with p110 to bind to p85, thereby inhibiting the phosphorylation of AKT (Thr308). CircSEMA4B inhibited the phosphorylation of AKT (Ser473) through miR-330-3p/PDCD4 axis. Taken together, circSEMA4B is a novel negative regulator of PI3K/AKT signaling pathway.

## Materials and methods

### Tissue samples

Tumor tissues and their adjacent normal tissues of 110 BC patients were collected from the Department of Breast and Thyroid Surgery of Shanghai Tenth People’s Hospital of Tongji University (Shanghai, China). Patient received radiotherapy, chemotherapy or any other neoadjuvant therapy before surgery were excluded. All tissue specimens were immediately snap-frozen in liquid nitrogen until further use. Our study protocols were approved by Institutional Ethics Committees of Shanghai Tenth People’s Hospital and informed consent was obtained from all patients or their relatives.

### Cell culture and transfection

The human embryonic kidney cell line HEK293T, human BC cell lines MDA-MB-231, HCC-1937, BT549, MCF-7, SKBR3, and the normal breast epithelial cell line MCF-10A were purchased from Chinese Academy of Sciences (Shanghai, China). All BC cell lines and HEK293T were cultured in Dulbecco’s modified Eagle’s medium (DMEM) (Gibco, USA) with 10% fetal bovine serum (FBS) (Gibco, USA). MCF-10A cells were cultured in mammary epithelial basal medium (MEBM) (Cambrex, USA). All cells were cultured at 37 °C with 5% CO_2_. Hieff Trans^TM^ Liposomal Transfection Reagent (Yeasen, China) was used for transfection according to the manufacturer’s instructions. Small, interfering, specifically targeting human circSEMA4B (si-circSEMA4B), non-specific negative control oligos (si-NC), circSEMA4B related constructs cloned on pcDNA 3.1(+) vector were purchased from IBSBio (Shanghai, China). Human miR-330-3p-mimics, non-specific negative control (miR-330-3p-NC), and miR-330-3p inhibitor were purchased from RiboBio (Guangzhou, China). The lentivirus carrying circSEMA4B-related constructs were constructed by ZORIN (Shanghai, China) and transfection procedures were performed according to the manufacturer’s instructions. We used DNA Midiprep Kits (Qiagen, Hilden, Germany) to prepare plasmid.

### RNA extraction and qRT-PCR

Total RNA was extracted from frozen tissues and cultured cells by Trizol reagent (Invitrogen, Carlsbad, CA, USA) and the concentration and purity of RNA samples was assessed with a Nanodrop 2000 spectrophotometer (Thermo Fisher Scientific, USA). Hifair® III 1st Strand cDNA Synthesis SuperMix (Yeasen, China) was used to reverse RNA into cDNA. Hieff® qPCR SYBR® Green Master Mix (Yeasen, China) was used for quantitative real-time polymerase chain reaction (qRT-PCR). Expression of circRNA, miRNA, and mRNA were assessed by threshold cycle (CT) values and analyzed using the 2^−ΔΔCt^ method. Primers designed in this study are shown in Supplementary Table S2.

### Confirming specificity for circSEMA4B

PCR products amplified by primers were separated on 1% agarose gel to verify the specificity of the circSEMA4B PCR products. Sanger sequencing was performed to validate the sequence of circSEMA4B.

### RNase R resistance analysis of circRNAs

MDA-MB-231 and MCF-7 cell lines were treated with RNase R (4 U/mg, Epicenter) and incubated for 30 min at 37 °C. Then, the treated RNAs were reverse transcribed with specific primers and detected by qRT-PCR assay.

### Actinomycin D assay

MDA-MB-231 and MCF-7 cells were treated with 2 mg/ml actinomycin D (Merck, Germany) to block transcription. The remaining RNAs extracted from treated cells was assessed by RT‐qPCR.

### Subcellular fractionation

Nuclear and Cytoplasmic Extraction Reagents (Thermo Fisher Scientific) were used for subcellular fractionation of BC cells. We used U6 as nuclear control and GAPDH as cytoplasmic control.

### FISH assay

Ribo™ Fluorescent in Situ Hybridization Kit (Ribo, China) was used in FISH assay. Specific probes for the circSEMA4B were designed and synthesized by RiboBio (Guangzhou, China) and specific probes for the miR-330-3p were designed and synthesized by IBSBio (Shanghai, China). 4’,6-Diamidino-2-Phenylindole (DAPI) was used to stain cell nuclei. Fluorescence microscope (Olympus BX53 Biological Microscope) was used to capture the images of cells.

### MTT assay

A density of 2000 cells per well were placed into 96-well plates. The cells were detected in accordance with the manufacturer’s instructions using MTT assay kit (Sigma, Santa Clara, CA, USA). The 490 nm optical density was detected by a microplate reader respectively at 24, 48, 72, and 96 h.

### Colony formation assay

A density of 1000 cells per well was transferred into six-well plates. When the colonies were visible after been cultured for about 10 days, cell colonies were washed twice by using cold phosphate buffered saline (PBS), fixed with 75% ethanol, and stained with 0.1% crystalline purple until the colonies were visible. Then colonies were counted and photographed.

### Wound healing assay

MDA-MB-231 cells were transfected with a range of constructs as indicated in 6-well plates. When the treated cells reached about 80% confluent, a scratch was produced in the cell monolayer by drawing a 200-μl-pipette tip over the surface of each well, holding the tip perpendicular to the plate. The monolayers were washed twice with 1x PBS and cultured with DMEM medium with 2%FBS. Wound healing was observed under a light microscope and pictures were taken at 0, 24, 36, and 48 h at the same position to observe cell movement.

### Transwell assays

Transwell chambers (Corning, Inc., Lowell, MA, USA) were used to measure the migration and invasion ability of the cells in 24-well plates.

Place desired number of Matrigel-coated inserts into 24-well plates and allow them to come to room temperature before performing invasion assays (This step is not required for migration assays). Cells were transferred into the upper chamber with 200 μl serum-free medium and medium with 10% FBS was added to the lower chamber. 12–24 h later, cells in the upper chamber were carefully removed by a cotton swab. Then, the cells on the opposite side of the filter were fixed with 70% ethanol for 30 min and stained with 0.1% crystal violet for 10 min. Representative pictures were taken with a microscope (Leica Microsystems, Mannheim, Germany) and migrated cells were counted in five random fields.

### RNA immunoprecipitation (RIP) and methylated RNA immunoprecipitation (MeRIP)

RIP and MeRIP was conducted using the BersinBio^TM^ RNA Immunoprecipitation Kit (Guangzhou, China) and BersinBio^TM^ Methylated RNA immunoprecipitation according to manufacturer’s instructions, respectively. Anti-AGO2 (Abclonal, China), Anti-m^6^A (Abcam, USA) and anti-IgG (Abclonal, China) were used. The extracted RNAs were analyzed by qRT-PCR.

### Dual-luciferase reporter assay

To confirm that miR-330-3p directly targets circSEMA4B, wild and mutant reporter plasmids of circSEMA4B were individually designed and synthesized by IBSBio (Shanghai, China). 293T cells were co-transected with the constructed reporter plasmids, together with miR-330-3p mimics or miR-330-3p-NC using Lipofectamine® 2000 (Invitrogen; Thermo Fisher Scientific, USA). 24–48 h later, luciferase activities were measured by the Dual-Luciferase® Reporter Assay kit (Yeasen, China). Then firefly to Renilla luciferase ratios were calculated.

### Western blotting analysis

Proteins were extracted by using RIPA lysis buffer (Beyotime, Jiangsu, China) and the concentrations were detected by using the protein assay kit (Beyotime, Jiangsu, China). Protein lysates were separated by 10% sodium dodecyl sulfate-polyacrylamide gels and then transferred to nitrocellulose membrane (Beyotime, Jiangsu, China), which was incubated 10 min with protein free rapid blocking buffer (Epizyme, Shanghai, China) and immunoblotted overnight at 4 °C with primary antibodies: anti-PDCD4 (1:1000, Bioworld, USA), anti-PTEN (1:1000, Santa Cruz Biotechnology, USA), anti-PI3K(p85) (1:1000, Abcam, USA), anti-PI3K(p110) (1:1000, Proteintech, USA), anti-AKT (1:1000, Proteintech, USA), anti-p-AKT (1:1000, Abcam, USA), anti-PDK1 (1:1000, Proteintech, USA), anti-p-PDK1 (1:1000, CST, USA), anti-SEMA4B-211aa (1:250, Abclonal, China), anti-Flag (1:1000, Abclonal, China), anti-SEMA4B (1:500, Absin, China) and anti-Actin (1:10,000, Abclonal, China). The next day, the membranes were incubated in secondary antibodies for 1 h at room temperature. Signals of protein bands were scanned by Odyssey Infrared scanning system (Li-Cor, Lincoln, NE, USA). The specific antibody against the SEMA4B-211aa peptide produced by circSEMA4B was generated by Abclonal (Wuhan, China). Through analyzing the specificity, hydrophilicity, and immunogenicity of the predicted translated peptide, the RARTHSATVKTTSRSSC segment was chosen as the antigenic region for the preparation of specific antibodies. The antibody was obtained by inoculating rabbits and purified using affinity chromatography columns. The concentration and titer of the specific antibody have been verified by Abclonal. Original western blots were shown in Supplementary Fig. S4.

### Co-immunoprecipitation (co-IP)

Cells were lysed in co-IP Lysis Buffer supplemented with protease and phosphatase inhibitors. The supernatant was collected and subjected to immunoprecipitation using indicated primary antibodies at 4 °C overnight. Then the lysates were incubated with 5 μl protein A and protein G agarose at 4 °C overnight. The collected agarose-protein complexes were centrifugated and washed with cold Wash buffer for 3 times and then subjected to SDS-PAGE, followed by Western blotting. The co-IP Reagent test kit (Absin, Shanghai, China) was purchased from Absin.

### GST pull-down assay

Cell lysates with GST-tagged SEMA4B-211aa and GST alone were incubated with Glutathione Sepharose 4B at 4 °C for 1 h, and the Sepharose were washed with washing buffer for 3 times. Glutathione Sepharose 4B binding with GST-tagged SEMA4B-211aa or GST were incubated with His-tagged PI3K(p85) at 4 °C overnight. Then the Sepharose complexes were centrifugated, washed, and then subjected to western blotting.

### Enzyme-linked immunosorbent assay (ELISA)

PI3K activity was determined by ELISA according to conversion of PIP2 lipid to PIP3 lipid. Cellular PIP2/PIP3 samples obtained from BC cells were mixed and incubated with PIP2/PIP3 Detector proteins, then added to a PIP2/PIP3-coated microplate for competitive binding. A peroxidase-linked secondary detection reagent was used to detect PIP2/PIP3 protein binding to the plate. The absorbance was measured at 450 nm, and the amount of PIP2/ PIP3 generated was calculated from a standard curve using known concentrations of the lipid product.

### Xenograft tumor assay

Athymic nude mice (4 weeks; 18–22 g) were ordered from the laboratory animal center of Shanghai. Approximately 1 × 10^6^ MDA-MB-231 cells with stable expression of Flag-circSEMA4B, Flag-circSEMA4B-MUT, Flag-circSEMA4B-NC, and Flag-circSEMA4B-211aa were injected into the second mammary fat of the mice (*n* = 4, each group). Then, tumor size was measured and calculated every week using the following formula: Volume (mm^3^) = 0.5 * width^2^ * length. After 5 weeks, the mice were killed by cervical dislocation and the tumors were collected. The animal protocols complied with the rule of the ethics committee of Tongji University.

### Immunohistochemistry (IHC)

Fresh tumor tissue samples from the nude mice were fixed in 4% paraformaldehyde, hydrated through ethanol solution, and embedded in paraffin. The paraffin-embedded tissue was sectioned into 4-μm slides, then the sections were incubated with anti-PDCD4, anti-PI3K(p110), and anti-p-AKT antibodies to measure PDCD4, PI3K(p110), and p-AKT expression. Images were captured under a microscope (Leica Microsystems, Mannheim, Germany) at the appropriate magnification.

### Statistical analysis

The significance of differences between groups was assessed by GraphPad Prism V8.0 (GraphPad, CA, USA). Comparisons between two groups were analyzed by Student’s *t* test. Relationships between the expression of circSEMA4B and various clinicopathological variables were analyzed by chi-square test and Fisher’s exact test. Comparisons between paired specimens were analyzed by Wilcoxon matched-pairs signed rank test. Data were obtained from three independent experiments which are presented as the means ± standard deviation (SD) and a *P*-value < 0.05 was considered significant.

## Supplementary information

Supplementary information

Supplementary information

Reproducibility checklist

## Data Availability

The PI3K/AKT signaling-associated genes were obtained from PathCards (https://pathcards.genecards.org/). The circRNAs sequence data were obtained from circbase (http://circrna.org/). All data included in this study are available upon request by contact with the corresponding author.
