# Supplementary material for: CircSEMA4B inhibits the progression of breast cancer by encoding a novel protein SEMA4B-211aa and regulating AKT phosphorylation
Source: Cell Death Dis. 2022 Sep 17;13(9):794. doi: 10.1038/s41419-022-05246-1 (PMC9482637; doi:10.1038/s41419-022-05246-1)

CircSEMA4B inhibits the progression of breast cancer by encoding a novel protein SEMA4B-211aa and regulating AKT Phosphorylation

Xuehui Wang[1,2], Wei Jian[1,2], Qifeng Luo[1] and Lin Fang[1]*

[1]Department of Thyroid and Breast Surgery, Shanghai Tenth People's Hospital, Tongji University School of Medicine, Shanghai 200072, China

[2] These authors contributed equally: Xuehui Wang, Wei Jian.

** Correspondence: 1200038@tongji.edu.cn*

Supplementary file 1：**Table S1** A gene list of 92 PI3K/AKT signaling-related genes and corresponding circRNAs (xlsx 27kb).

Supplementary file 2：**Table S2** Primers and siRNAs used in this study.

Supplementary file 3：**Figure S1 a** circAKT2 expression in 15 paired BC tissues and adjacent normal tissues. **b** circDEPTOR expression in 15 paired BC tissues and adjacent normal tissues. **c** circFOXO3 expression in 15 paired BC tissues and adjacent normal tissues. **d** circGIGYF2 expression in 15 paired BC tissues and adjacent normal tissues. **e** circSEMA4B expression in 15 paired BC tissues and adjacent normal tissues. **f** circWDR59 expression in 15 paired BC tissues and adjacent normal tissues. **g** circYAP1 expression in 15 paired BC tissues and adjacent normal tissues.

Supplementary file 4: **Figure S2 a** The concentration and titer of anti-SEMA4B-211aa was verified by Dot Immunobinding Assay. **b-d** m^6A modification sites were predicted in circSEMA4B sequence according to SRAMP. **e** MeRIP was performed and the co-precipitated RNA was subjected to RT-qPCR for circSEMA4B.

Supplementary file 5：**Figure S3 a** The mRNA level of SEMA4B was detected in BC cells transfected with si-circSEMA4B and LV-circSEMA4B. **b** The protein level of SEMA4B was detected in BC cells transfected with si-circSEMA4B and LV-circSEMA4B. **c** Expression of p110 in BC cells transfected with Flag-circSEMA4B, Flag-circSEMA4B-MUT, Flag-circSEMA4B-NC and Flag-circSEMA4B-211aa. **d** Co-IP silver staining was used to demonstrate the potential proteins interacting with SEMA4B-211aa. **e** p110 could not be detected in the anti-SEMA4B-211aa precipitates by Co-IP. **f** Prokaryotic purified His-tagged-p85, GST-tagged-SEMA4B-211aa and

GST was subjected to GST pull down. **g** Expression of PDCD4 was detected in BC cells transfected with Flag-circSEMA4B, Flag-circSEMA4B-MUT, Flag-circSEMA4B-NC and Flag-circSEMA4B-211aa. **H** Expression of SEMA4B-211aa was detected in BC cells transfected with si-PDCD4.

Supplementary file 6：Figure S4 Original western blots.

Table S2 Primers and siRNAs used in this study

Gene	Sequences
18S-F	5'-TAGAGGGACAAGTGGCGTTC-3'
18S-R	5'-CGCTGAGCCAGTCAGTGT-3'
circSEMA4B-F	5′-GCCCTGGTGGTTGATGG-3′
circSEMA4B-R	5′-CTTTGGCTCCGCGAGAT-3′
GAPDH-F	5'-CAGGAGGCATTGCTGATGAT-3'
GAPDH-R	5'-GAAGGCTGGGGCTCATTT-3'
SEMA4B-F	5′-GATTCGAAGCTGAACACATCTC-3′
SEMA4B-R	5′-GTTGCTACTGAGTGCAAAGAG-3’
PDCD4-F	5’-AGTGACGCCCTTAGAAGTGG-3'
PDCD4-R	5'-TCATATCCACCTCCTCCACA-3'
PI3K(p110)-F	5’-GAGTACCTTGTTCCAATCCCAG-3’
PI3K(p110)-R	5’-TTCCTCTTTAGCACCCTTTCG-3’
miR-330-3p-F	5’-GCAGAGATTCCGTTGTCGT-3’
miR-330-3p-R	5’-GCGAGCACAGAATTAATACGAC-3’
U6-F	5'-CAAATTCGTGAAGCGTTCCATAT-3'
U6-R	5'-GCTTCACGAATTTGCGTGTCATCCTTGC-3'
si-PDCD4-Sense	5'-CCAUUACUCUAGACCAAAUTT-3'
si-PDCD4- Anti-sense	5'-AUUUGGUCUAGAGUAAUGGTT-3'
si-circSEMA4B-1-Sense	5’-GCAUCUGCAAGGCUCUGAAGATT-3’
si-circSEMA4B-1-Anti-sense	5’-UCUUCAGAGCCUUGCAGAUGCTT-3’
si-circSEMA4B-2-Sense	5’-CCGCAUCUGCAAGGCUCUGAATT-3’
si-circSEMA4B-2-Anti-sense	5’-UUCAGAGCCUUGCAGAUGCGGTT-3’
si-circSEMA4B-3-Sense	5’-UCUGCAAGGCUCUGAAGAGCGTT-3’
si-circSEMA4B-3-Anti-sense	5’-CGCUCUUCAGAGCCUUGCAGATT-3’
si-NC- Sense	5’-UUCUCCGAACGUGUCACGUTT-3’
si-NC- Anti-sense	5’-ACGUGACACGUUCGGAGAATT-3’

Figure S1

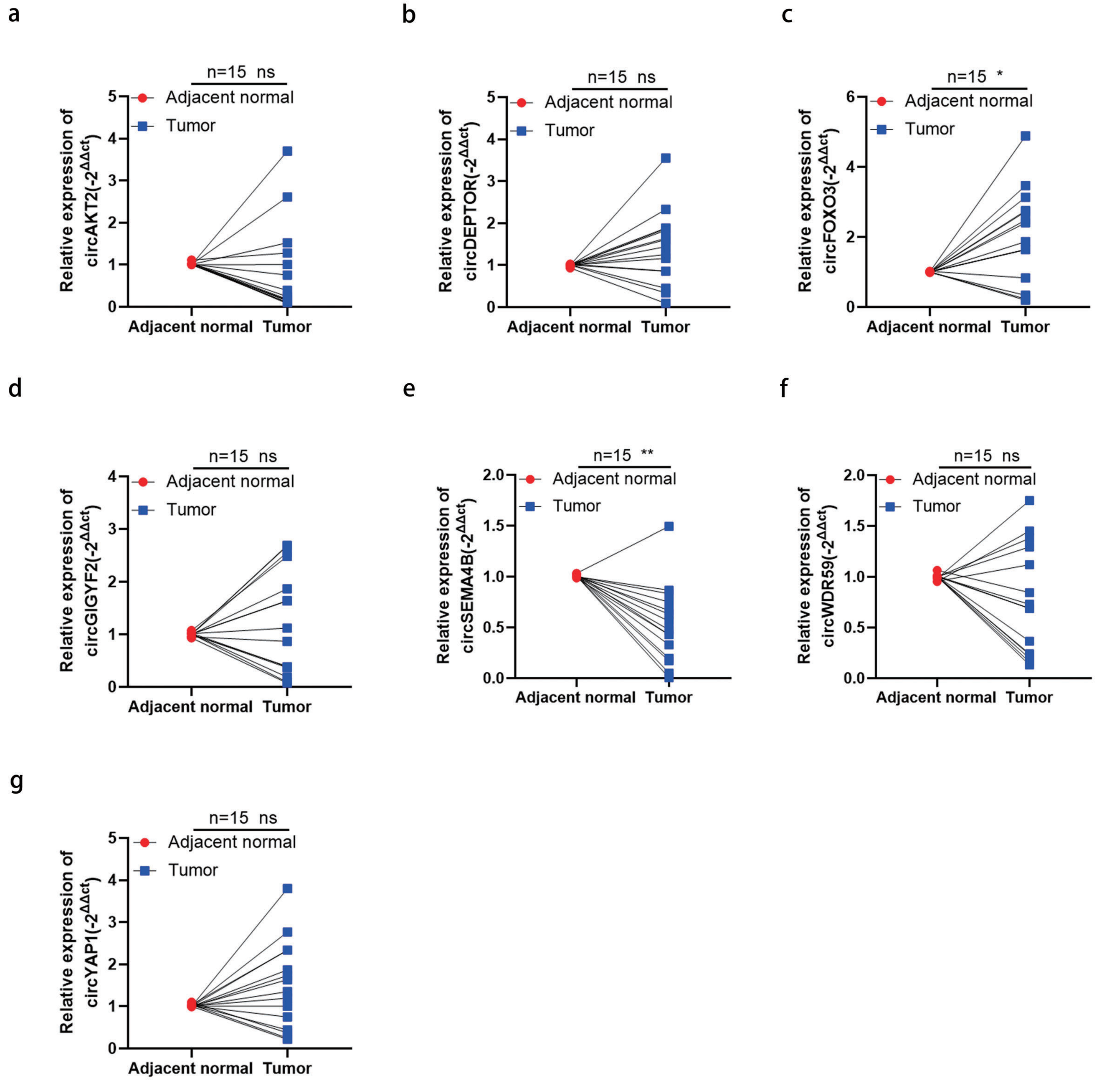
a
n=15 ns
Adjacent normal
Tumor
Relative expression of circAKT2(-2^ΔΔct)
b
n=15 ns
Relative expression of circDEPTOR(-2^ΔΔct)
c
n=15 *
Relative expression of circFOXO3(-2^ΔΔct)
d
n=15 ns
Relative expression of circGIGYF2(-2^ΔΔct)
e
n=15 **
Relative expression of circSEMA4B(-2^ΔΔct)
f
n=15 ns
Relative expression of circWDR59(-2^ΔΔct)
g
n=15 ns
Relative expression of circYAP1(-2^ΔΔct)

Figure S2

a

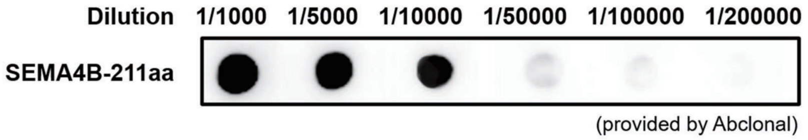

b

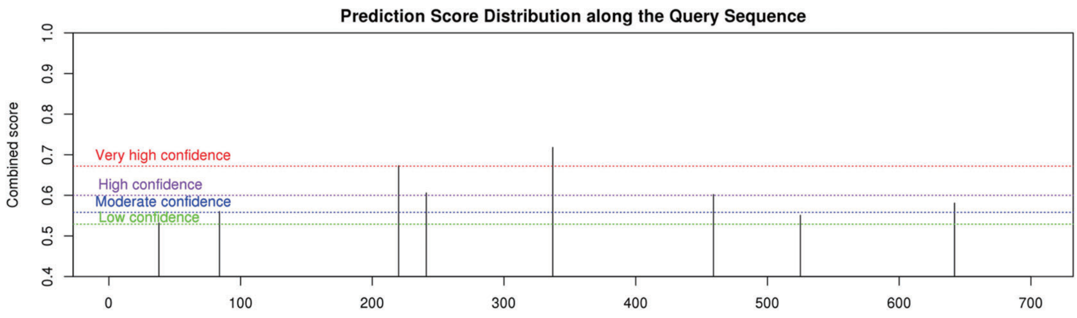

c

#	Position	Sequence context	Structural context	Score(binary)	Score(knn)	Score(spectrum)	Score(combined)	Decision
1	38	CAUUC CUCAG AUUCG<br>AAGCU GAACA CAUCU<br>CCAAC UACAC AGCCC	PMMMM MPPPP HHHHH<br>HHHPP PPMMM MMPPP<br>PMMMM MMMMM MPPPI	0.455	0.397	0.656	0.532	m^6A site (**Low** confidence)
2	84	UCUGC UGAGC AGGGA<br>UGGCA GGACC CUGUA<br>CGUGG GUGCU CGAGA	PPPPP HHHPP PPPPI<br>IPPPM PIPPP PHHHH<br>HHHPP PPIPM MPPPP	0.491	0.355	0.680	0.560	m^6A site (**Moderate** confidence)
3	220	CAGUG CAGCU UCAAG<br>GGCAA GGACC CACAG<br>CGCGA CUGUC AAAAC	PPPPP PBBBB BBBBP<br>PPPPP PPPBB BBBPP<br>PPPPP PPPPM MMMMM	0.612	0.764	0.746	0.673	m^6A site (**Very high** confidence)
4	241	GACCC ACAGC GCGAC<br>UGUCA AAACU ACAUC<br>AAGAU CCUCC UGCCG	PPBBB BBPPP PPPPP<br>PPPMM MMMMM MMMMM<br>MMMMM MMMMP PPPPP	0.491	0.621	0.762	0.606	m^6A site (**High** confidence)
5	337	UGUAC CUACA UCAAC<br>AUGGA GAACU UCACC<br>CUGGC AAGGG ACGAG	PHHHH HHHHH HHHPP<br>PPPPM MMMMM MMPPP<br>IIPPP PIIPP PPMMP	0.697	0.796	0.738	0.718	m^6A site (**Very high** confidence)
6	459	UGAUG GCGAG CUCUA<br>CACUG GAACA GUCAG<br>CAGCU UCCAA GGGAA	PPPMP PPIIP PPIII<br>PPPIP PMPPP PPPBP<br>PIPPP PPPMM PPPPM	0.481	0.471	0.785	0.602	m^6A site (**High** confidence)
7	525	CCAAA GCCUU CGCCC<br>CACCA AGACC GAGAG<br>CUCCC UCAAC UGGCU	IIPPP PMMMP PPPPI<br>IIIPP PPIPP IPPBP<br>PPPMM MMMMM MPPPI	0.430	0.393	0.736	0.551	m^6A site (**Low** confidence)
8	642	GAUCU ACUUU UUCUU<br>CAGCG AGACU GGCCA<br>GGAAU UUGAG UUCUU	HPPPM MPPPP IIPPP<br>PBPPP PPPBP PPPPI<br>PPMMM MMMMP PPPHH	0.545	0.521	0.639	0.581	m^6A site (**Moderate** confidence)

d

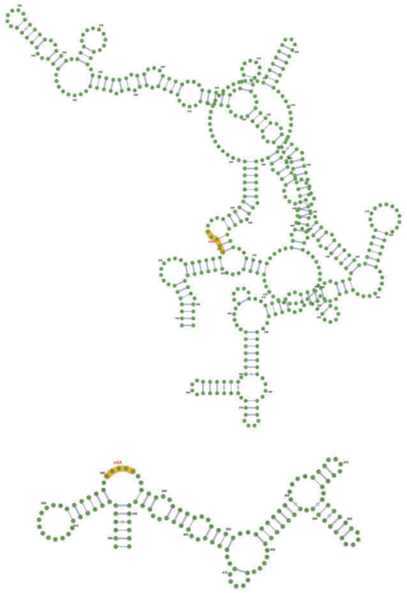

e

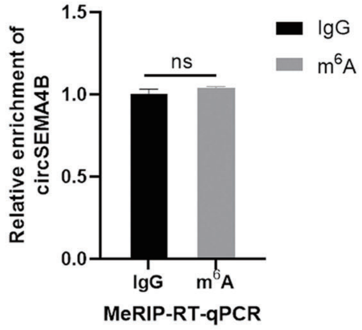

Figure S3

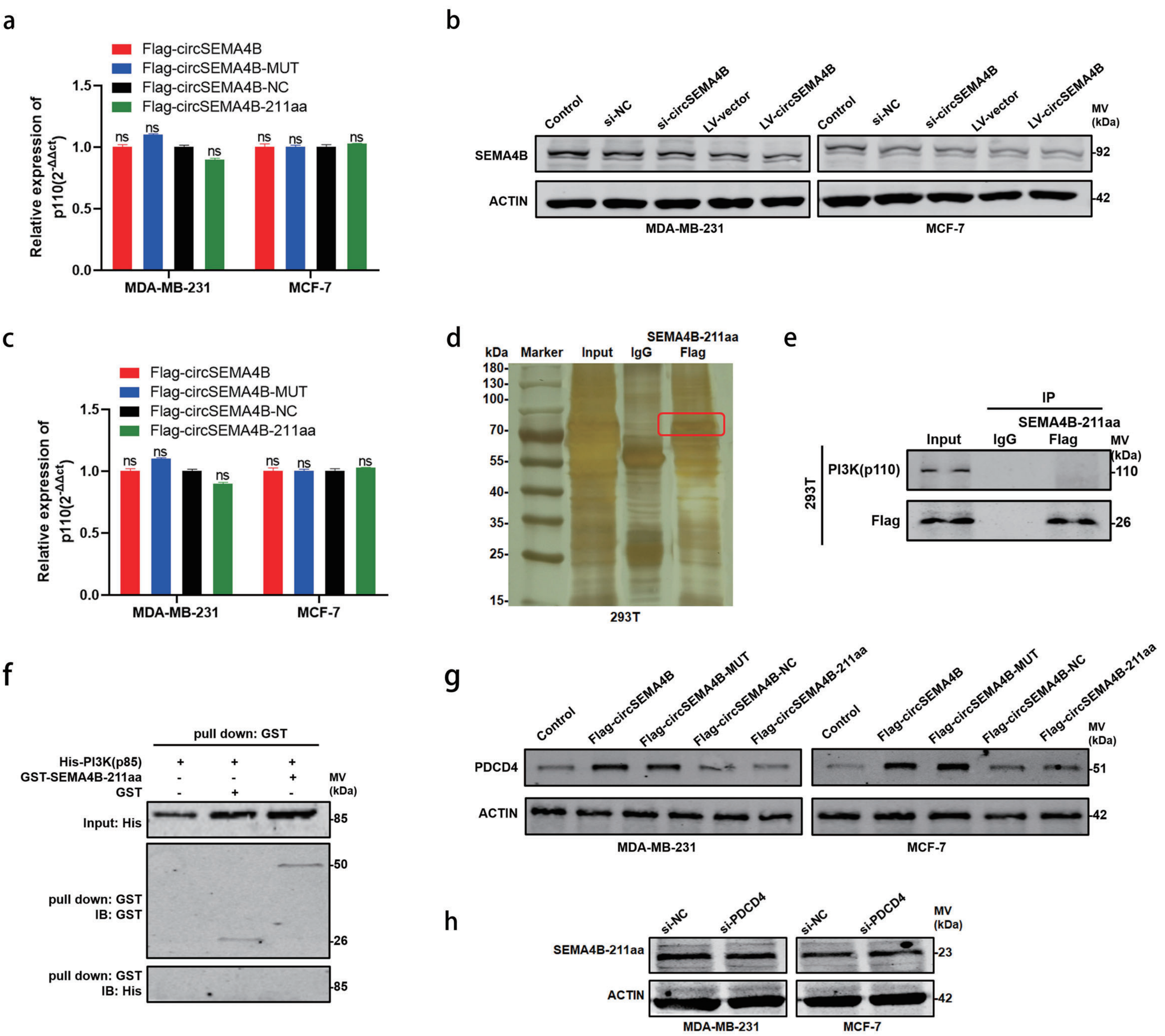
a
Flag-circSEMA4B
Flag-circSEMA4B-MUT
Flag-circSEMA4B-NC
Flag-circSEMA4B-211aa
Relative expression of p110(2^-ΔΔct)
ns
MDA-MB-231
MCF-7
b
Control
si-NC
si-circSEMA4B
LV-vector
LV-circSEMA4B
MV (kDa)
SEMA4B
ACTIN
92
42
MDA-MB-231
MCF-7
c
Flag-circSEMA4B
Flag-circSEMA4B-MUT
Flag-circSEMA4B-NC
Flag-circSEMA4B-211aa
Relative expression of p110(2^-ΔΔct)
ns
MDA-MB-231
MCF-7
d
kDa
Marker
Input
IgG
SEMA4B-211aa Flag
180
130
100
70
55
40
35
25
15
293T
e
IP
SEMA4B-211aa
Input
IgG
Flag
MV (kDa)
293T
PI3K(p110)
Flag
110
26
f
pull down: GST
His-PI3K(p85)
GST-SEMA4B-211aa
GST
MV (kDa)
Input: His
pull down: GST IB: GST
pull down: GST IB: His
85
50
26
85
g
Control
Flag-circSEMA4B
Flag-circSEMA4B-MUT
Flag-circSEMA4B-NC
Flag-circSEMA4B-211aa
MV (kDa)
PDCD4
ACTIN
51
42
MDA-MB-231
MCF-7
h
si-NC
si-PDCD4
MV (kDa)
SEMA4B-211aa
ACTIN
23
42
MDA-MB-231
MCF-7

Figure S4 Original western blots.

Figure.3f

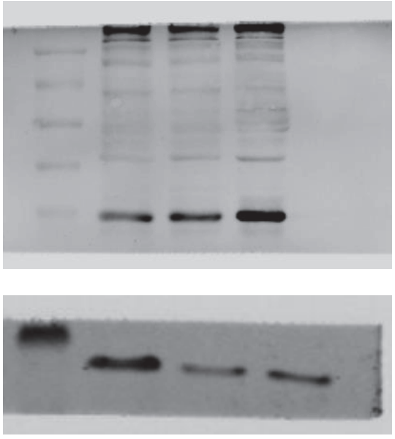

Figure.3g

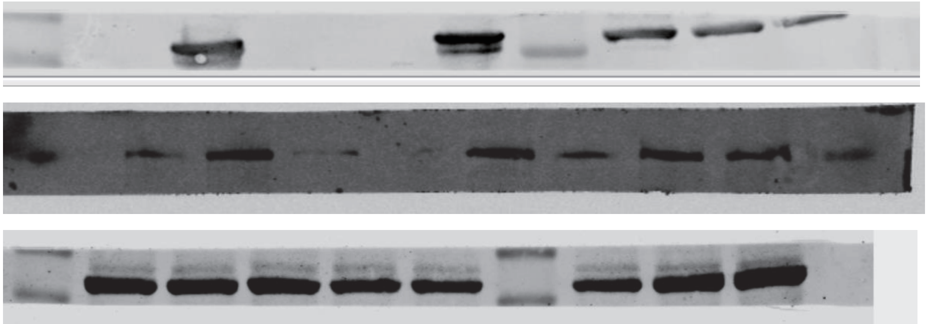

Figure.3h

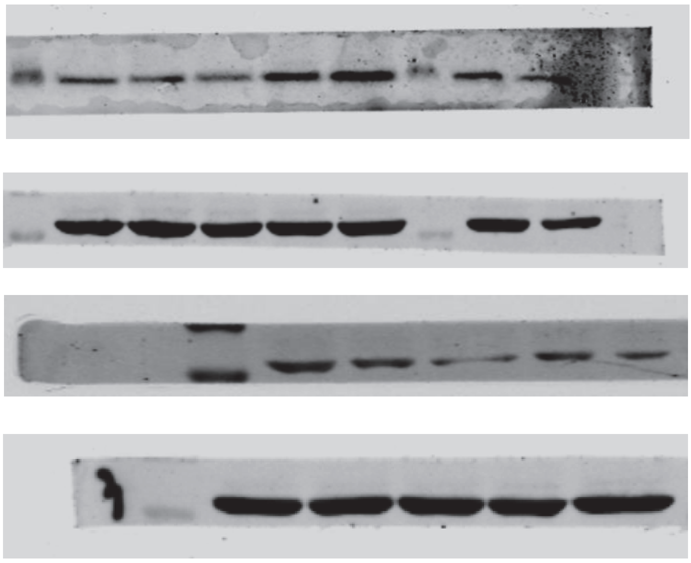

Figure.4a

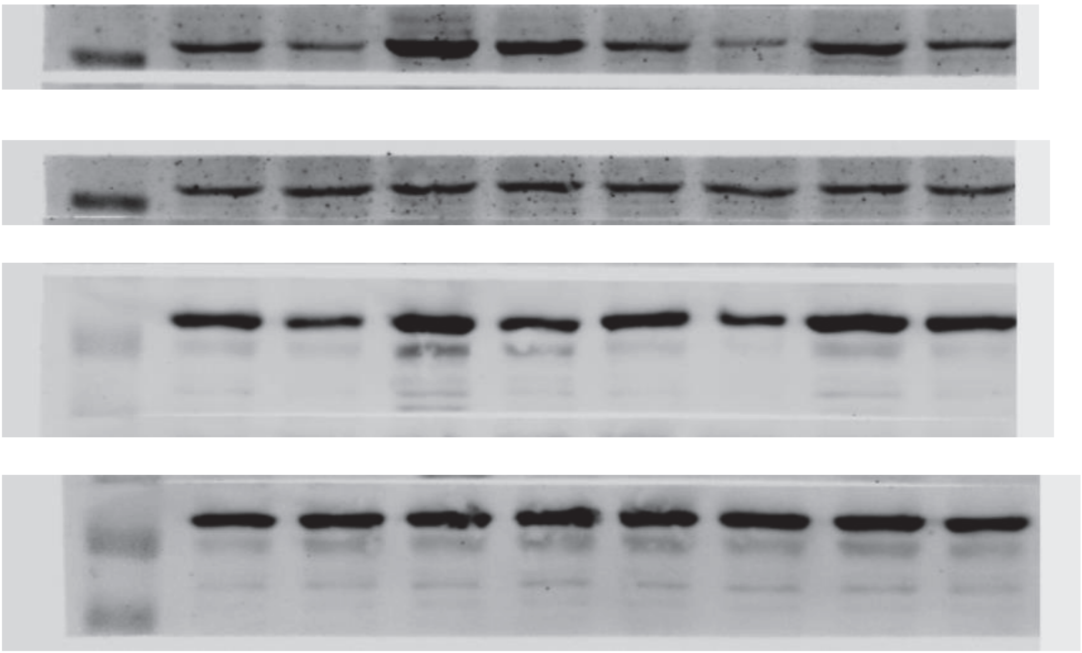

Figure.4b

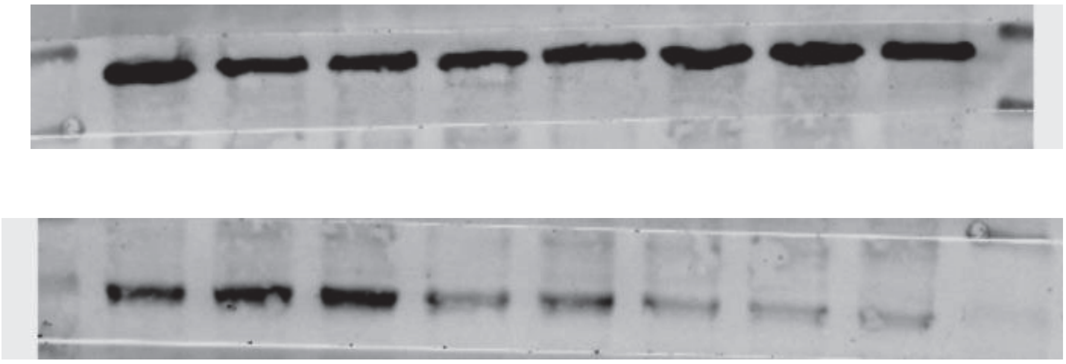

Figure.5a

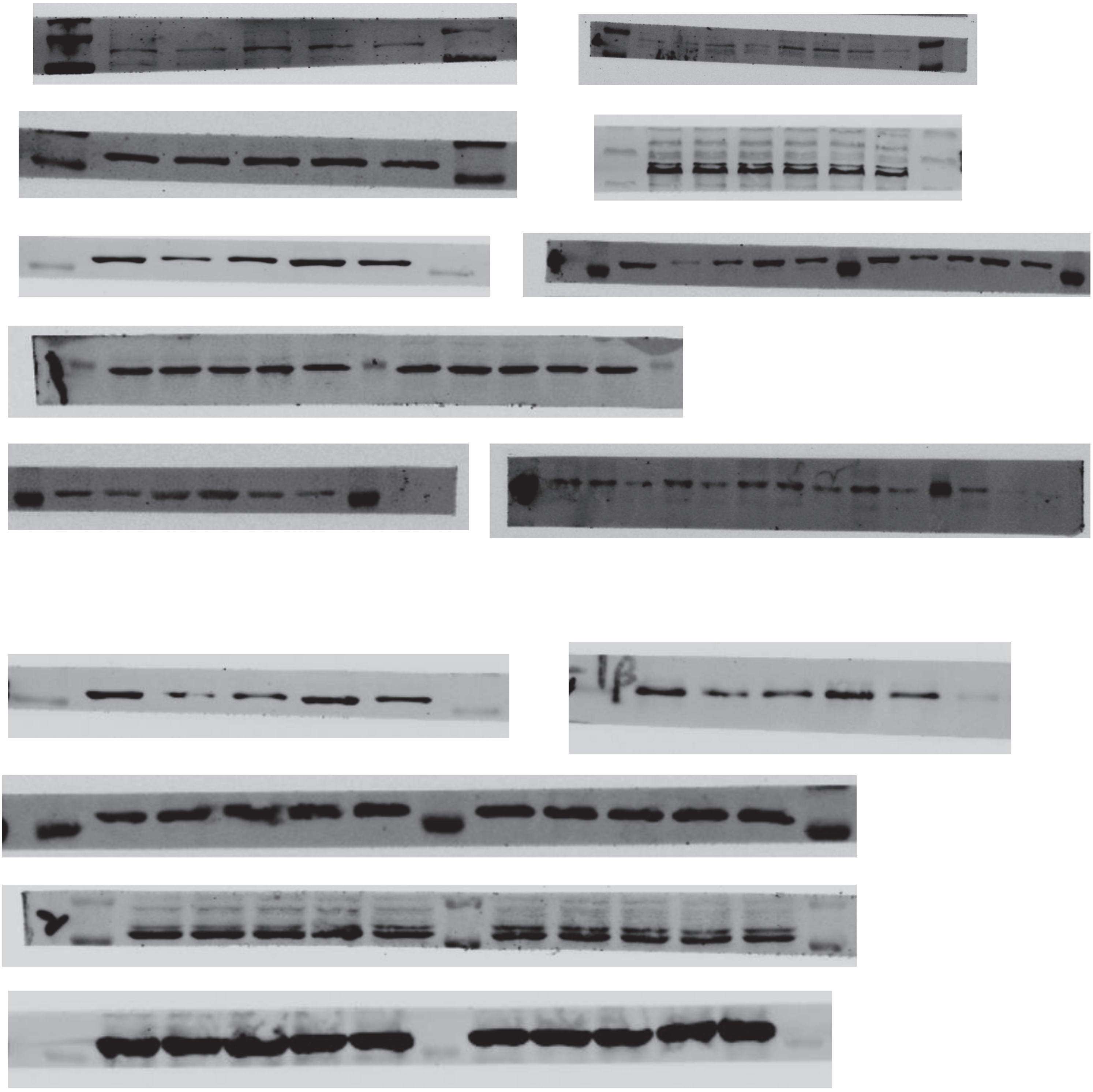

Figure.5b

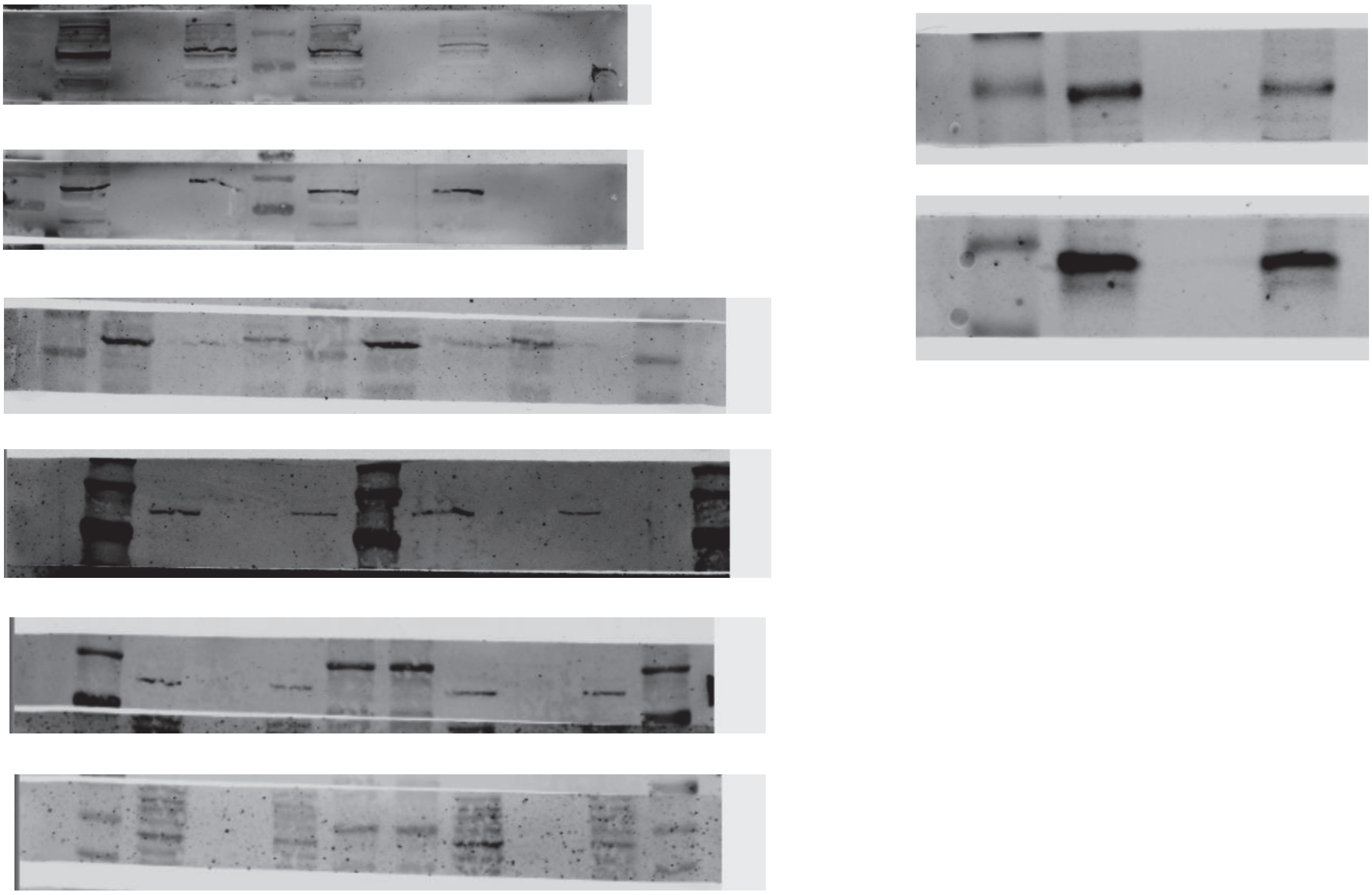

Figure.5c

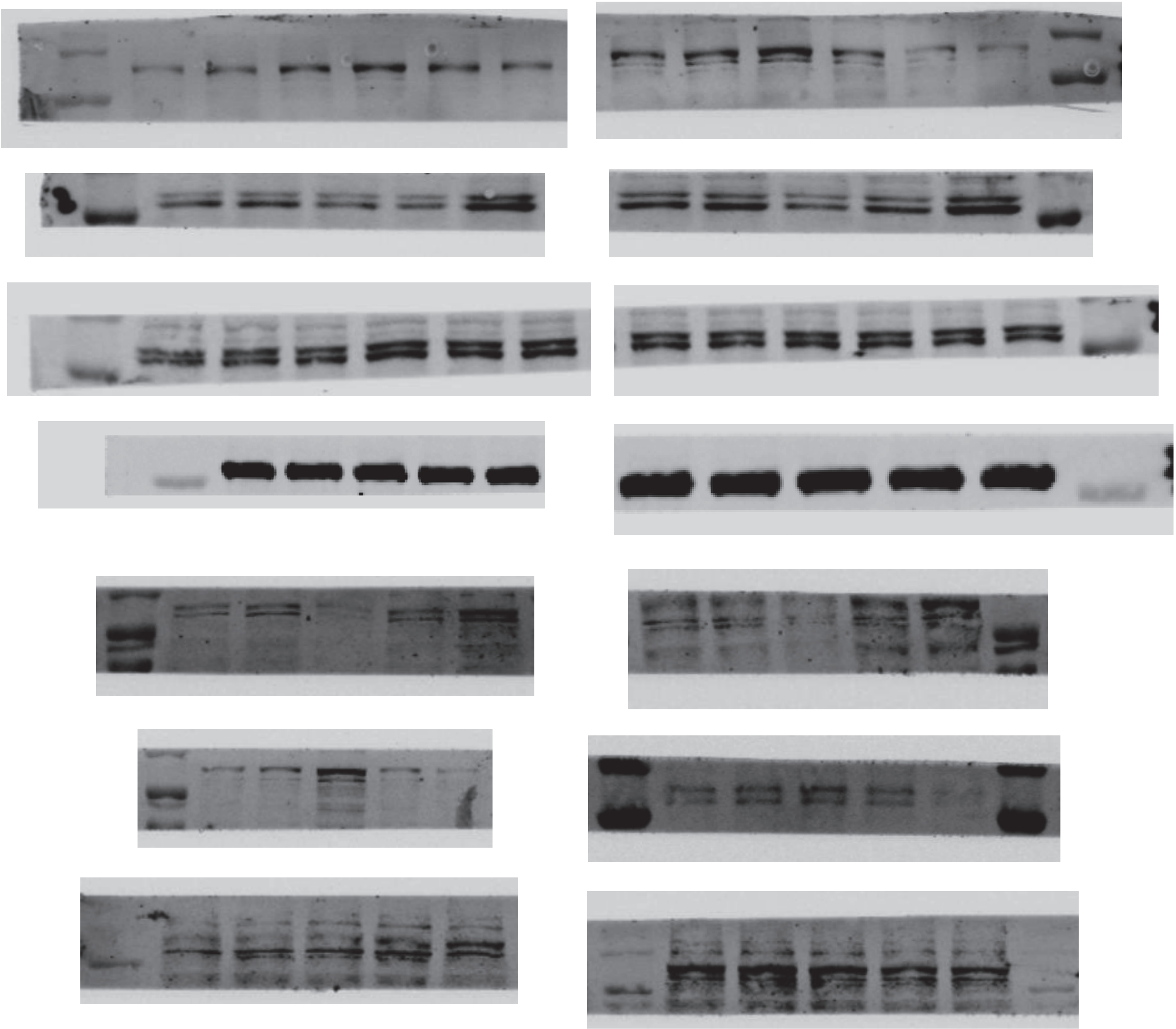

Figure.6J

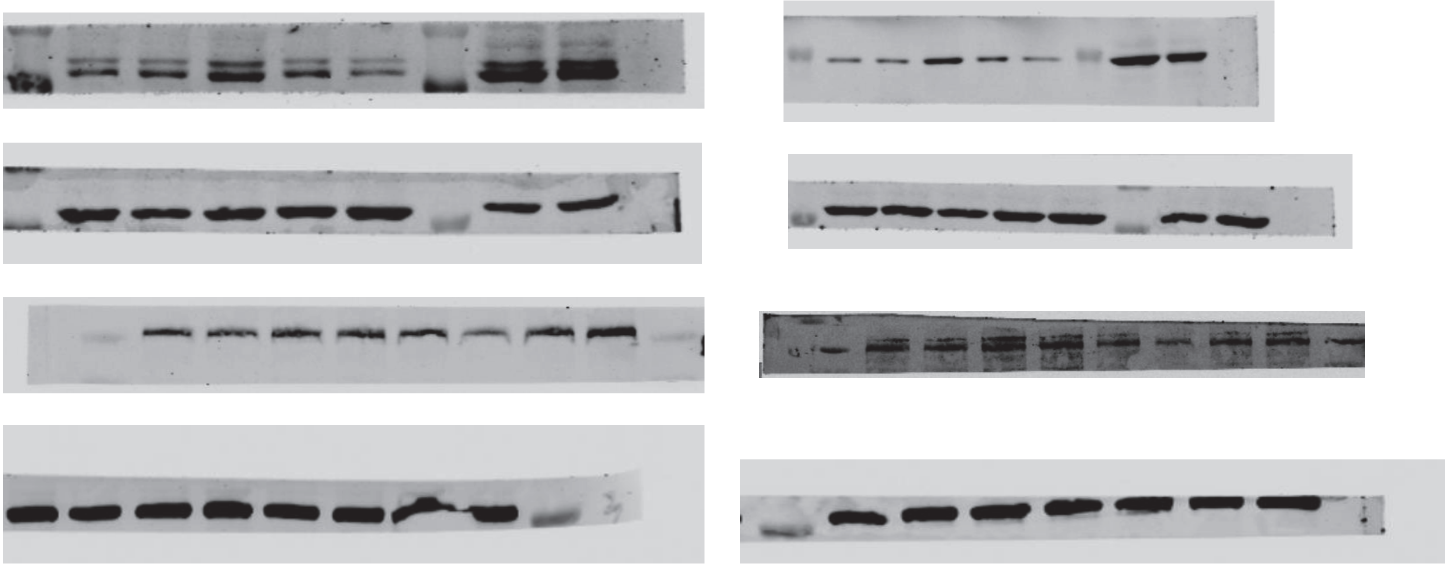

Figure.7e

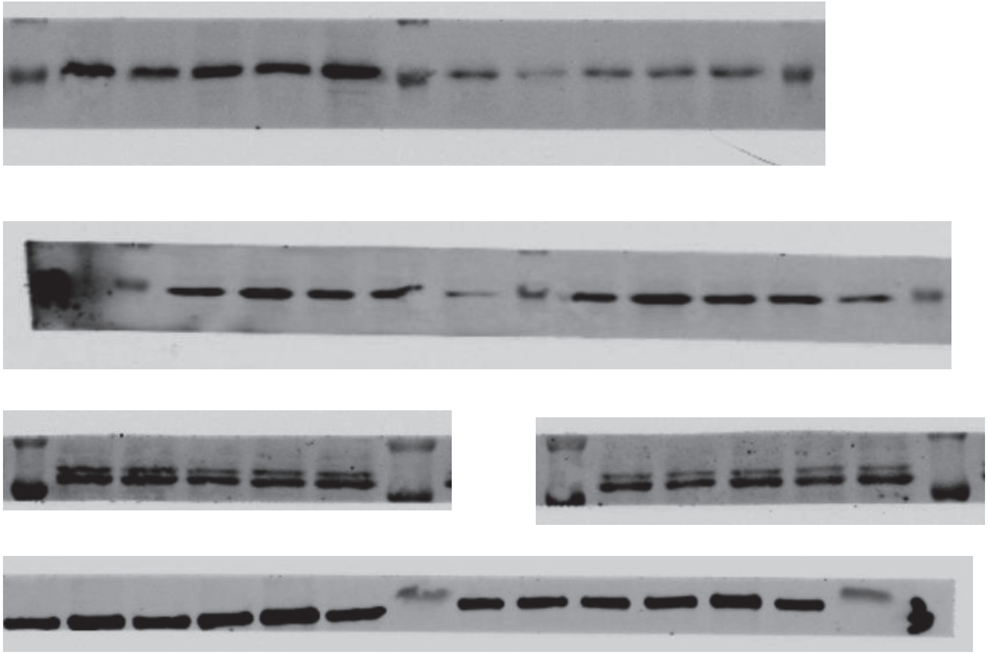

Figure.8b

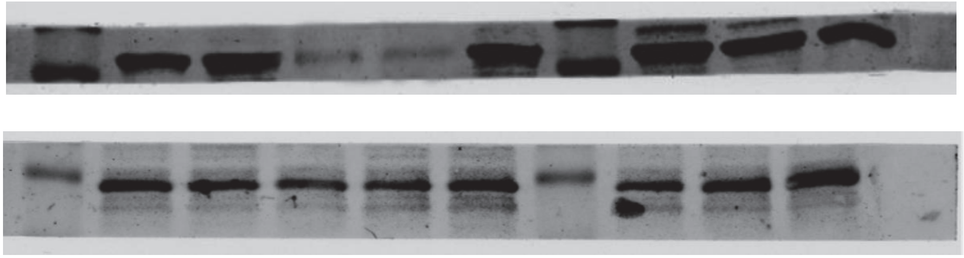

Figure.S3b

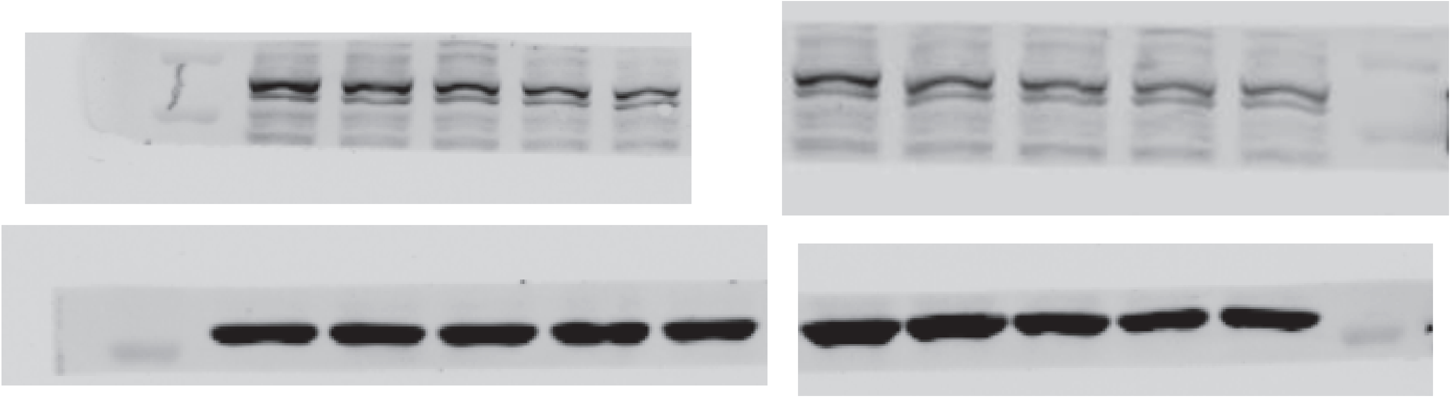

Figure.S3e

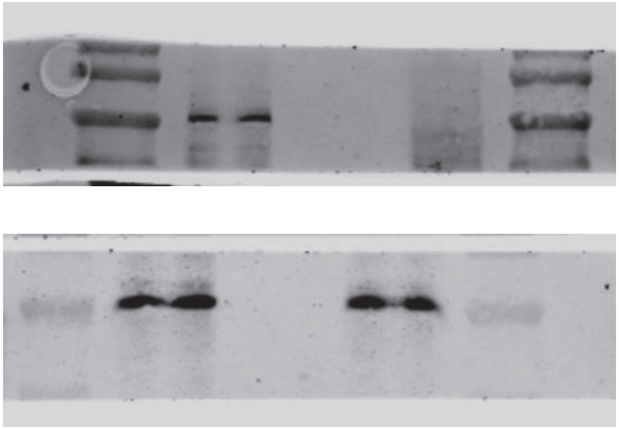

Figure.S3f

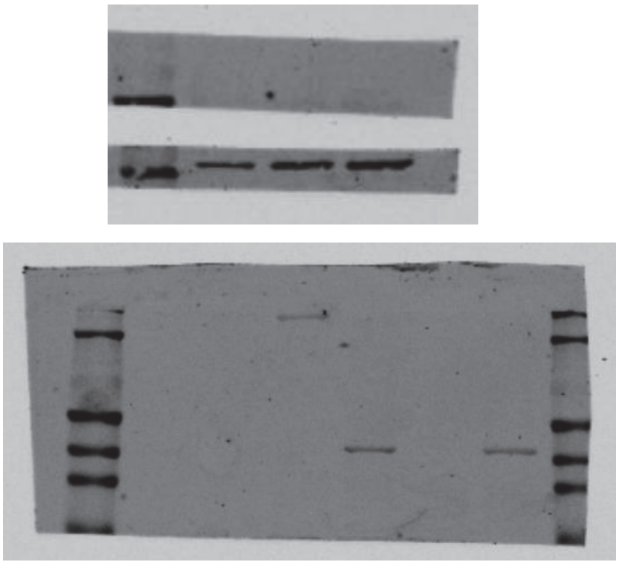

Figure.S3g

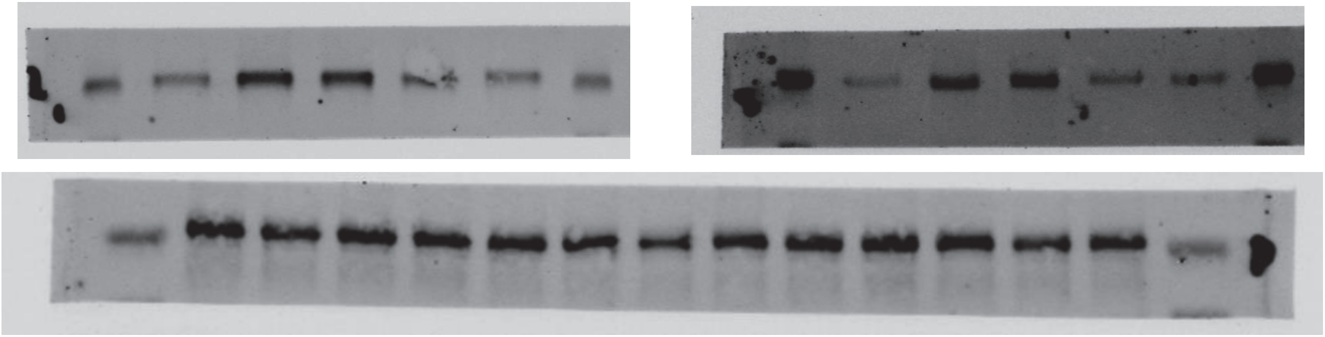

Figure.S3h

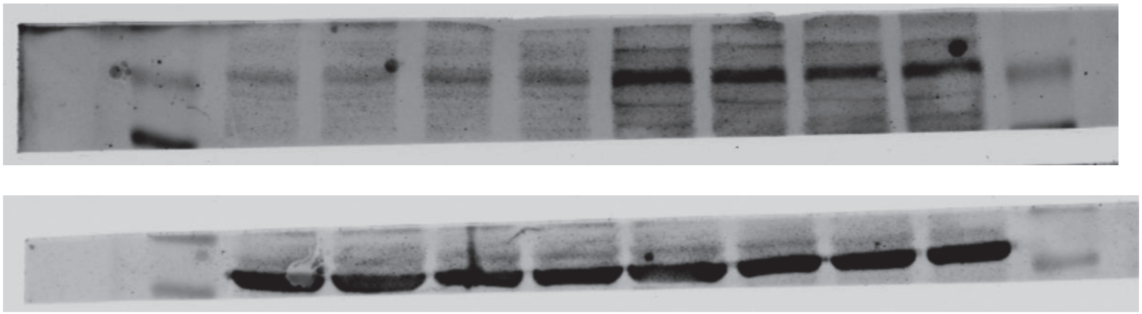

Supplement: Supplementary file 1 — Supplementary information [file 41419_2022_5246_MOESM1_ESM.pdf]
